# Supplementary material for: Air pollution, physical activity and ischaemic heart disease: a prospective cohort study of interaction effects
Source: BMJ Open. 2021 Apr 13;11(4):e040912. doi: 10.1136/bmjopen-2020-040912 (PMC8051414; doi:10.1136/bmjopen-2020-040912)
Supplement: Supplementary data [file bmjopen-2020-040912supp001.pdf]

A1: Distributions of PM10 and PM2.5 moving averages (lag 1-5) for the person-years included in the study

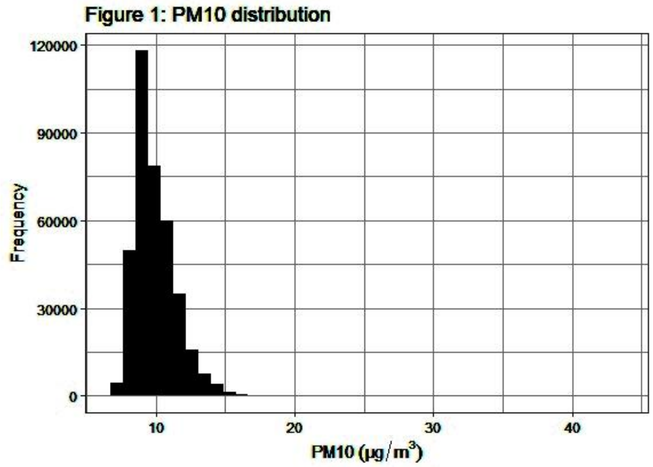

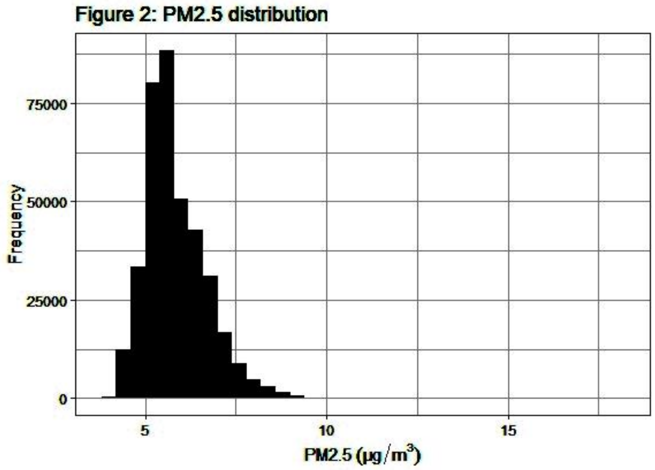

A2: Distributions of PM10 and PM2.5 moving averages (lag 1-5) for the person-years included in the study at different levels of physical activity (exercise in training clothes).

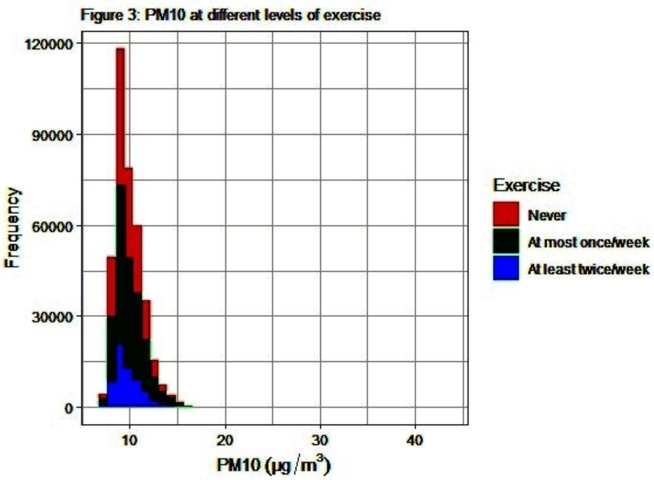

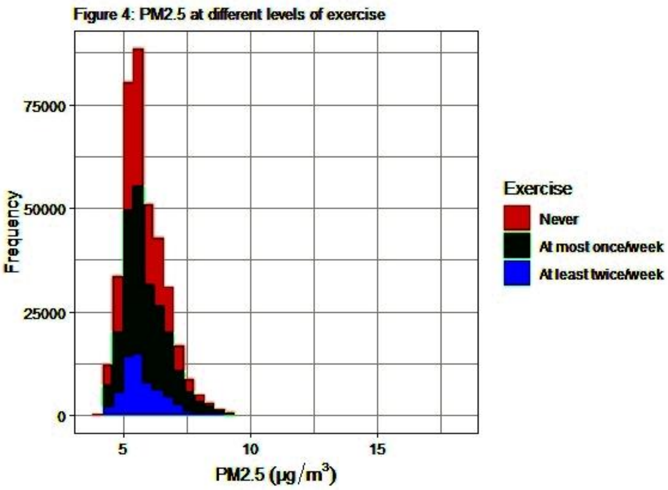

B: Sensitivity analysis by excluding the individuals with follow-up time below the 25th percentile

Table 1. Hazard ratios (95% CI) for IHD associated with different exercise and commuting habits among persons with different air pollution exposure at home addresses.

Exercise in training clothes	Overall model with no interaction effects	Adjusted[a] HRs in categories of high and low particle exposure		Adjusted[a] interaction hazard ratio
		Low PM_{10}[b]	High PM_{10}[b]	Benefits of exercise/commuting comparing high and low particle exposure
Never	1	1	1	
≤ once/week	1.00 (0.84-1.19)	1.01 (0.84-1.21)	1.02 (0.85-1.23)	1.02 (0.79-1.31)
≥ twice/week	0.88 (0.76-1.02)	1.0 (0.77-1.31)	0.48 (0.34-0.7)	0.48 (0.31-0.76)
		Low PM2.5[c]	High PM2.5[c]	
Never		1	1	
≤ once/week		1.01 (0.84-1.21)	1.02 (0.85-1.23)	1.01 (0.78-1.31)
≥ twice/week		0.95 (0.72-1.24)	0.53 (0.37-0.75)	0.56 (0.36-0.87)
Active commuting per season		Low PM10[b]	High PM10[b]	
Non-active commuting	1	1	1	
≤ two seasons of four	1.02 (0.89-1.16)	1.16 (0.92-1.46)	0.85 (0.66-1.09)	0.73 (0.52-1.03)
> two seasons of four	0.74 (0.6-0.92)	0.91 (0.74-1.12)	0.83 (0.68-1.02)	0.91 (0.69-1.2)
		Low PM2.5[c]	High PM2.5[c]	
Non-active commuting		1	1	
≤ two seasons of four		1.18 (0.93-1.48)	0.84 (0.65-1.08)	0.70 (0.50-0.98)
> two seasons of four		0.98 (0.80-1.2)	0.79 (0.64-0.96)	0.82 (0.62-1.09)

[a]Adjusted for sex, calendar year, education, smoking, alcohol intake, occupation, neighbourhood mean income, leisure time physical activity, active commuting

[b]Low PM_{10}: ≤9.6 μg/m³; High PM_{10}: >9.6 μg/m³

[c]Low $PM_{2.5}$ ≤5.7 μg/m³; High $PM_{2.5}$: >5.7 μg/m³

Table 2. Hazard ratios (95% CI) for IHD associated with high air pollution levels (vs low) at home address among persons with different exercise/commuting habits.

	Overall model with No interaction effects	Adjusted[a] HRs in different exercise categories		
		Exercise in training clothes		
		Never	≤ once/week	≥ twice/week
Low PM_{10}[b]	1	1	1	1
High PM_{10}[b]	1.22 (0.96-1.56)	1.25 (1.0-1.56)	1.27 (1.03-1.58)	0.61 (0.40-0.93)
Low $PM_{2.5}$[c]	1	1	1	1
High $PM_{2.5}$[c]	0.95 (0.74-1.21)	1.15 (0.92-1.43)	1.16 (0.94-1.44)	0.64 (0.42-0.97)
		Active commuting		
		Non-active commuting	≤ two seasons of four	> two seasons of four
Low PM_{10}[b]		1	1	1
High PM_{10}[b]		1.28 (1.04-1.57)	0.94 (0.68-1.29)	1.16 (0.9-1.5)
Low $PM_{2.5}$[c]		1	1	1
High $PM_{2.5}$[c]		1.23 (1.0-1.51)	0.88 (0.64-1.21)	0.99 (0.76-1.27)

[a]Adjusted for sex, calendar year, education, smoking, alcohol intake, occupation, neighbourhood mean income, leisure time physical activity, active commuting

[b]Low PM_{10}: ≤9.6 μg/m³; High PM_{10}: >9.6 μg/m³

[c]Low $PM_{2.5}$ ≤5.7 μg/m³; High $PM_{2.5}$: >5.7 μg/m³

C: Sensitivity analyses with PM10 and PM2.5 concentrations categorized by tertile limits

Table 3. Hazard ratios (95% CI) for IHD associated with different exercise and commuting habits among persons with different air pollution exposure at home addresses.

Exercise in training clothes	Overall model with no interaction effects	Proportional hazard p[d]-value*	Adjusted[a] HRs in categories of high and low particle exposure					
			Low PM_{10}[b]	Proportional hazard p-value[d]	Moderate PM_{10}[b]	Proportional hazard p-value[d]	High PM_{10}[b]	Proportional hazard p-value[d]
Never	1		1		1			
≤ once/week	1.03 (0.90-1.16)	0.36	0.93 (0.74-1.16)	0.42	1.15 (0.94-1.41)	0.95	0.99 (0.80-1.23)	0.37
≥ twice/week	0.76 (0.62-0.93)	0.09	0.95 (0.68-1.32)	0.54	0.83 (0.60-1.15)	0.16	0.51 (0.34-0.77)	0.86
			Low $PM_{2.5}$[c]		Moderate $PM_{2.5}$[c]		High $PM_{2.5}$[c]	
Never			1		1			
≤ once/week			0.98 (0.78-1.22)	0.67	1.08 (0.88-1.32)	0.60	1.02 (0.82-1.26)	0.48
≥ twice/week			0.91 (0.65-1.27)	0.71	0.83 (0.60-1.14)	0.13	0.54 (0.36-0.82)	0.60

[a]Adjusted for sex, calendar year, education, smoking, alcohol intake, occupation, neighbourhood mean income, leisure time physical activity, and active commuting

[b]Low PM_{10}: ≤9.6 µg/m^3; High PM_{10}: >9.6 µg/m^3

[c]Low $PM_{2.5}$ ≤5.7 µg/m^3; High $PM_{2.5}$: >5.7 µg/m^3

[d]p-value of the Schoenfeld residual test of proportional hazards

Table 4. Hazard ratios (95% CI) for IHD associated with high and moderate air pollution levels (vs low) at home address among persons with different exercise/commuting habits.

	Overall model with no interaction effects	Proportional hazard p-value[d]	Adjusted[a] HRs in categories of high and low particle exposure					
			Exercise in training clothes					
			Never	Proportional hazard p-value[d]	≤ once/week	Proportional hazard p-value[d]	≥ twice/week	Proportional hazard p-value[d]
Low PM_{10}[b]	1		1		1		1	
Moderate PM_{10}[b]	1.04 (0.81-1.34)	0.55	1.05 (-0.83 - 1.32)	0.35	1.30 (-1.03 1.63)	0.95	0.92 (0.61-1.39)	0.26
High PM_{10}[b]	1.00 (0.69-1.45)	0.99	1.16 (-0.87 1.53)	0.13	1.23 (0.93-1.64)	0.13	0.62 (0.37-1.04)	0.56
Low $PM_{2.5}$[c]	1		1		1		1	
Moderate PM_{10}[c~~b~~]	1.12 (0.88-1.43)	0.94	1.11 (-0.89 1.39)	0.73	1.23 (-0.98-1.53)	0.73	1.02 (0.67-1.54)	0.30
High $PM_{2.5}$[c]	1.15 (0.79-1.68)	0.13	1.18 (-0.89 1.57)	0.05	1.23 (0.92-1.64)	0.04	0.71 (0.42-1.18)	0.22

[a]Adjusted for sex, calendar year, education, smoking, alcohol intake, occupation, neighbourhood mean income, leisure time physical activity, and active commuting

~~[b]Low PM_{10}: ≤9.6 µg/m³; High PM_{10}: >9.6 µg/m³~~

~~[c]Low $PM_{2.5}$ ≤5.7 µg/m³; High $PM_{2.5}$: >5.7 µg/m³~~

[b]Low PM10: 7.2 - 9.1 µg/m3; Moderate PM10: 9.2 - 10.3 µg/m3; High PM10: 10.4 - 43.0 µg/m3

[c]Low PM2.5 : 4.1 - 5.4 µg/m3; Moderate PM2.5: 5.5 - 6.1 µg/m3; High PM2.5: 6.2 - 17.9 µg/m3

[d]p-value of the Schoenfeld residual test of proportional hazards

Table 5. Interaction hazard ratios between exercise and air pollution for incident IHD

Exercise in training clothes	Adjusted[a] interaction hazard ratio			
	Benefits of exercise comparing moderate~~high~~ with low PM_{10}[b] exposure		Benefits of exercise comparing high with low PM_{10}[b] ~~particle~~ exposure	
	Moderate PM_{10}[b]	Proportional hazard p-value[d]	High PM_{10}[b]	Proportional hazard p-value[d]
Never	1		1	
≤ once/week	1.24 (0.91-1.68)	0.51	1.07 (0.78-1.45)	0.99
≥ twice/week	0.88 (0.55-1.39)	0.58	0.54 (0.32-0.92)	0.81
	Benefits of exercise comparing moderate~~high~~ with low $PM_{2.5}$[c] exposure		Benefits of exercise comparing High~~moderate~~ with low $PM_{2.5}$[c] exposure	
Never	1		1	
≤ once/week	1.11 (0.82-1.50)	0.97	1.04 (0.77-1.42)	0.87
≥ twice/week	0.92 (0.58-1.46)	0.45	0.60 (0.35-1.02)	0.87

[a]Adjusted for sex, calendar year, education, smoking, alcohol intake, occupation, neighbourhood mean income, leisure time physical activity, and active commuting

~~[b]Low PM_{10}: ≤9.6 µg/m³; High PM_{10}: >9.6 µg/m³~~

~~[c]Low $PM_{2.5}$ ≤5.7 µg/m³; High $PM_{2.5}$: >5.7 µg/m³~~

[b]Low PM10: 7.2 - 9.1 µg/m3; Moderate PM10: 9.2 - 10.3 µg/m3; High PM10: 10.4 - 43.0 µg/m3

[c]Low PM2.5 : 4.1 - 5.4 µg/m3; Moderate PM2.5: 5.5 - 6.1 µg/m3; High PM2.5: 6.2 - 17.9 µg/m3

[d]p-value of the Schoenfeld residual test of proportional hazards
