# Supplementary material for: Air pollution, physical activity and ischaemic heart disease: a prospective cohort study of interaction effects
Source: BMJ Open. 2021 Apr 13;11(4):e040912. doi: 10.1136/bmjopen-2020-040912 (PMC8051414; doi:10.1136/bmjopen-2020-040912)
Supplement: Author's manuscript [file bmjopen-2020-040912.draft_revisions.pdf]

BMJ Open is committed to open peer review. As part of this commitment we make the peer review history of every article we publish publicly available.

When an article is published we post the peer reviewers' comments and the authors' responses online. We also post the versions of the paper that were used during peer review. These are the versions that the peer review comments apply to.

The versions of the paper that follow are the versions that were submitted during the peer review process. They are not the versions of record or the final published versions. They should not be cited or distributed as the published version of this manuscript.

BMJ Open is an open access journal and the full, final, typeset and author-corrected version of record of the manuscript is available on our site with no access controls, subscription charges or pay-per-view fees (http://bmjopen.bmj.com).

If you have any questions on BMJ Open's open peer review process please email info.bmjopen@bmj.com

BMJ Open

Air pollution, physical activity and ischemic heart disease - A prospective cohort study of interaction effects

Journal:	*BMJ Open*
Manuscript ID	bmjopen-2020-040912
Article Type:	Original research
Date Submitted by the Author:	25-May-2020

Complete List of Authors:	Raza, Wasif; Umeå Universitet Medicinska fakulteten, Public Health and Clinical Medicine, Sustainable Health<br>Krachler, Benno; Umeå Universitet Medicinska fakulteten, Public Health and Clinical Medicine, Sustainable Health<br>Forsberg, Bertil; Umeå Universitet Medicinska fakulteten, Public Health and Clinical Medicine, Sustainable health<br>Sommar, Johan; Umeå Universitet Medicinska fakulteten, Public Health and Clinical Medicine, Sustainable health

Keywords:	Ischaemic heart disease < CARDIOLOGY, PUBLIC HEALTH, Coronary heart disease < CARDIOLOGY

SCHOLARONE™
Manuscripts

I, the Submitting Author has the right to grant and does grant on behalf of all authors of the Work (as defined in the below author licence), an exclusive licence and/or a non-exclusive licence for contributions from authors who are: i) UK Crown employees; ii) where BMJ has agreed a CC-BY licence shall apply, and/or iii) in accordance with the terms applicable for US Federal Government officers or employees acting as part of their official duties; on a worldwide, perpetual, irrevocable, royalty-free basis to BMJ Publishing Group Ltd ("BMJ") its licensees and where the relevant Journal is co-owned by BMJ to the co-owners of the Journal, to publish the Work in this journal and any other BMJ products and to exploit all rights, as set out in our licence.

The Submitting Author accepts and understands that any supply made under these terms is made by BMJ to the Submitting Author unless you are acting as an employee on behalf of your employer or a postgraduate student of an affiliated institution which is paying any applicable article publishing charge ("APC") for Open Access articles. Where the Submitting Author wishes to make the Work available on an Open Access basis (and intends to pay the relevant APC), the terms of reuse of such Open Access shall be governed by a Creative Commons licence – details of these licences and which Creative Commons licence will apply to this Work are set out in our licence referred to above.

Other than as permitted in any relevant BMJ Author's Self Archiving Policies, I confirm this Work has not been accepted for publication elsewhere, is not being considered for publication elsewhere and does not duplicate material already published. I confirm all authors consent to publication of this Work and authorise the granting of this licence.

For peer review only

Air pollution, physical activity and ischemic heart disease - A prospective cohort study of interaction effects

Wasif Raza [1,*], Benno Krachler [1], Bertil Forsberg [1] and Johan Nilsson Sommar [1]

[1] Department of Public Health and Clinical Medicine, Section of Sustainable Health, Umeå University, Sweden; wasif.raza@umu.se (W.R.); benno.krachler@umu.se (B.K.); bertil.forsberg@umu.se (B.F.); johan.sommar@umu.se (J.N.S.)

* Correspondence: wasif.raza@umu.se ; Tel.: +46-72-289-5144

For peer review only

Abstract

Objective: To assess a possible interaction effect between physical activity and air pollution on first incidence of ischemic heart disease (IHD).

Design: Prospective cohort study

Setting: Umeå, Northern Sweden

Participants: We studied 34,748 adult participants of Västerbotten Intervention Programme cohort from 1990 to January 2014. Annual particulate matter concentrations ($PM_{2.5}$ and PM_{10}) at the participants' residential addresses were modelled and a questionnaire on frequency of exercise and active commuting was completed at baseline. Cox proportional hazards modelling was used to estimate 1) association with physical activity at different levels of air pollution, and 2) the association with particulate matter at different levels of physical activity.

Outcome: First incidence of IHD.

Results: Over a mean follow-up of 12.4 years, there were 1,148 IHD cases. Overall, we observed an increased risk of IHD among physically active individuals with higher concentrations of particles at their home address. Exercise at least twice a week was associated with a lower risk of IHD among participants with high residential $PM_{2.5}$ (0.60; 95% CI: 0.44-0.82) and PM_{10} (0.55; 95% CI: 0.4-0.76). The same beneficial effect was not observed with low residential $PM_{2.5}$ (0.94; 95% CI: 0.72-1.22) and PM_{10} (0.99; 95% CI: 0.76-1.29). An increased risk associated with higher long-term exposure to particles was only observed among participants never exercising in training clothes and among those not performing any active commuting. However, only the interaction effect on hazard ratios for exercise was statistically significant.

Conclusion: Exercise was associated with a lower risk of first incidence of IHD among individuals with higher residential particle concentrations. An air pollution-associated risk was only observed among those who exercised less. The findings support the promotion of physical activity and a mitigation of air pollution.

Key Words: Air pollution, $PM_{2.5}$, PM_{10}, exercise, interaction, ischemic heart disease

For peer review only

Strengths and limitations of this study

- This study simultaneously evaluated the impact of physical activity and air pollution and their interaction on first incident IHD cases in a population with relatively low level of air pollution concentrations.
- For air pollution exposure, this study used individual time varying exposures of annual mean concentrations during follow-up based on population address registries.
- Another strength is the prospective design and the availability of baseline data on several important confounders.
- No exposure-response assessment could be performed since the statistical power only allowed for the formation of two exposure categories.
- Differences in air pollution exposure during active commuting might cause possible bias estimates due to exposure misclassification.

For peer review only

1 Introduction

Cardiovascular disease (CVD) is the most important cause of morbidity and premature mortality worldwide, accounting for 422.3 million cases and 17.92 million deaths in 2015 [1]. There is solid evidence that inflammation is a key upstream pathogenic mechanism [2]. Ambient air pollution, generally comprising ozone, sulphur dioxide, nitrogen dioxide and particulate matter (PM), is a leading contributor to the global burden of disease and an important risk factor for morbidity and mortality. Particulate matter is often measured as $PM_{2.5}$, to represent particles with a diameter of 2.5 micrometres or less, and PM_{10}, to represent particles with a diameter of 10 micrometres or less. Ambient $PM_{2.5}$ exposure alone has been estimated to account for 4.2 million deaths in 2015, of which 1.5 million deaths were caused by ischemic heart diseases (IHD) [3]. The underlying mechanisms mediating the pathogenic impact of air pollution involve systemic oxidative stress and inflammation [4,5]. Physical activity is a salutogenic factor in numerous non-communicable chronic diseases (NCDs), with physical inactivity being responsible for 6% of the burden of disease from coronary heart disease [6,7]. The beneficial effects of physical activity include protection against low-grade inflammation by releasing anti-inflammatory substances, such as interleukin 6, from contracting muscles [8,9].

Rapid urbanization and increased use of motorized transport contribute to modern day problems such as traffic congestion, traffic related air pollution and lack of physical activity. Promotion of active transportation by changing mode of transport from car to cycling and walking are among the different strategies used to tackle these challenges [10–12]. As inflammation is a causative mechanism for cardiovascular disease, it is conceivable that the anti-inflammatory effects of physical activity may mitigate the harmful effects associated with exposure to air pollution. However, one major concern with physical activity in a polluted environment is the increased inhalation of particles due to an increase in respiratory volume that may counteract the beneficial effects of physical activity [13,14].

The long-term effects of air pollution among individuals with different levels of leisure time physical activity have been estimated within the Danish Diet, Cancer, and Health cohort. The incidence of diabetes was assessed in relation to leisure time physical activity and nitrogen dioxide (NO_2) concentration at the home address. Residential NO_2 was found to be associated with increased incidence of diabetes, but only among physically active individuals [15]. As far as we know, only one study has examined the modifying effect of air pollution on the association between physical activity and cardiovascular disease. In their recent study, Kubesch and colleagues conclude that physical activity reduced the risk of first incidence of myocardial infarction (MI) and recurrent MI among individuals with high NO_2 concentration at the residential addresses [16]. As only one pollutant (NO_2) and only one cardiovascular outcome (MI) have been studied, the knowledge of a possible interaction between air pollution and physical activity on CVD is inconclusive.

A Taiwanese study found an independent inverse association of habitual physical activity with inflammation across different levels of $PM_{2.5}$ exposure, although long term exposure was associated with increased inflammation at all levels of physical activity [17].

We therefore aimed to examine interaction effects between physical activity and long-term exposure to $PM_{2.5}$ and PM_{10} at residential addresses on the incidence of ischemic heart disease (IHD). We wanted to

assess: 1) whether air pollution modifies the beneficial effects of physical activity on IHD, and 2) whether physical activity modifies the harmful effects of air pollution on IHD.

2 Methods

To determine the interaction effect between air pollution and physical activity on IHD incidence, we combined cohort data which comprised risk factors for IHD, national registry data on IHD incidence from the Swedish National Board of Health and Welfare, and yearly annual mean air pollution particle concentrations at the individuals' residential addresses using dispersion models from the Swedish Clean Air and Climate Research Program (SCAC).

VIP (Västerbotten Intervention Programme) is a population based screening and prospective cohort study, developed to reduce the risk of future CVD and diabetes by promoting a healthy lifestyle among individuals living in the Västerbotten region. The SCAC developed methods to estimate exposure to source-specific particulate matter such as $PM_{2.5}$ and PM_{10} and at residential addresses in Gothenborg, Stockholm and Umeå.

2.1 Study population

VIP is an ongoing population-based health investigation survey of all individuals at ages 40, 50 or 60 years, depending upon risk factors, living in the Västerbotten region, who are invited to participate in systematic risk factor screening and individual counselling about healthy lifestyle habits. A detailed description of VIP has been presented elsewhere [18]. Between 1990 and 2014, a total of 42,488 of the VIP participants that lived in Umeå Municipality during the study period were included in the analysis. After exclusion of 7,740 participants with missing information on exercise, the study sample thus consisted of 34,748 individuals, 53% men and 47% women, 40-60 years of age at baseline examination, with no previous history of IHD at time of enrolment. After exclusion of individuals with missing information on included confounders the final number of included individuals were 31424 and 29218 for the analyses of exercise in training clothes and active commuting, respectively. All participants in the VIP gave their informed written consent, and the study was approved by the Regional Ethics Review Board at Umea University (DNR: 2014-136-32M and 2015/16-31Ö).

2.2 Leisure time exercise and active commuting

The VIP questionnaire includes various self-reported information on physical activity including frequency of leisure time physical activity and active commuting (cycling or walking to and from work). The association with IHD was assessed in relation to frequency of exercise in training clothes and amounts of active commuting. Exercise during the previous three months was categorized as never, rarely, once per week, 2-3 times per week or more than 3 times per week. Based on this information, participants were categorized as "Twice per week or more" if they exercised with a frequency of 2-3 times per week and more, "At most once a week" if they exercised rarely or once per week, and "Never" if no activity was performed. For active commuting, participants were asked about their mode of transport to work each season. Participants were classified in three categories: "Non- active commuting" if commuting every

For peer review only

season by car or bus, "At most two seasons out of four" if cycling or walking at most half a year and "More than two seasons out of four", if cycling and walking more than half a year.

2.3 Covariates

The VIP questionnaire also gathered information on participants' educational status, occupation, smoking, alcohol intake and economic status. Education was defined according to the International Standard Classification of Education (ISCED), United Nations Educational, Scientific and Cultural Organization (UNESCO) 1997. Participants were asked about the highest level education they had achieved with eight predefined categories ranging from "pre-school" to "university education." Alcohol intake was assessed by the reported frequency of consumption with answering options that ranged from "Never" to "2-4 times/week." Information on smoking was gathered by using the question, "How often do you smoke?" Information on occupational status was obtained with a question, "What kind of job do you have nowadays?" with the answering options of eight predefined categories. Finally, information on occupation status was asked with the question, "What is your current occupation?" with 8 categories ranging from permanently employed to retired.

2.4 Air pollution concentrations

Annual mean total concentrations of $PM_{2.5}$ and PM_{10} for the years 1990-2011 (and thereafter linearly extrapolated up until year 2014) were obtained from the SCAC research program, described in detail elsewhere [19]. Briefly, concentrations of $PM_{2.5}$ and PM_{10} were estimated within SCAC by applying dispersion models on local or regional emission inventories. These emission inventories contain detailed information on emissions from different source categories, such as road traffic exhaust, road traffic non-exhaust, domestic heating, shipping and industrial activities. For Umeå, inventories were validated through monitoring for consistency. Emissions from small-scale residential heating were assessed using registry data from chimney sweepers that included the type of wood stove or boiler. The addresses of these residences were geocoded using the geographical centre coordinate of the estate. Road traffic emission factors for PM exhaust for different vehicle types, speeds and driving conditions were calculated based on the Handbook Emission Factors for Road Transport (HBEFA) version 3.1 [20]. Estimates for non-exhaust contribution from brake and tire wear were based on Omstedt et al. [21]. The annual average emission from shipping was used in the modelling on a 1x1 km^2 grid resolution. The emissions from other sources such as industrial processes, off-road machinery and agriculture was collected from Swedish Meteorological and Hydrological Institute (SMHI). To obtain annual average emissions of $PM_{2.5}$ and PM_{10}, Gaussian models included in the Airviro air quality management system (SMHI, 2010) were used for simulation based on hourly meteorological data for 1990, 2000 and 2011 [22]. The comparison between measured and modelled $PM_{2.5}$ and PM_{10} agreed well at most monitoring stations (r^2=0.87 and r^2=0.65, respectively).

2.5 Outcome

For peer review only

We linked the records in VIP and through the unique Swedish personal identification number with data on first IHD event cases from the National Patient Register and the Cause of Death Register, both at the National Board of Health and Welfare, using primary discharge diagnoses for IHD according to International Classification of Diseases, 10th revision (ICD-10): code I20-125.

2.6 Participants and public involvement

There was no participants and public involvement in conducting or reporting our research.

2.7 Statistical Analysis

We performed survival analyses using Cox regression with age as underlying time scale to estimate hazard ratios (HRs), and 95% confidence intervals (CIs), to estimate 1) the association between first incident IHD and air pollution exposure at different levels of physical activity and 2) the association between first incident IHD and physical activity at different levels of air pollution exposure. Follow-up started at date of recruitment to the cohort and ended with the earliest of the date of first IHD case, emigration, death, or 31 December 2013. Interaction between physical activity and air pollution and their impact on IHD was studied by introducing an interaction term into the model. Residential annual mean particle concentrations were categorized as below or above the median concentration for $PM_{2.5}$ and PM10, respectively. Interaction with physical activity was assessed based on 1) the frequency of exercise in training clothes and 2) the number of seasons the individual walked or cycled to work. Active commuting by walking or cycling was categorized into three groups: non-active commuters, active commuters at most half of the year (up to two out of four seasons), active commuters more than half a year (more than two out of four seasons). Estimates were adjusted for: calendar year as a penalized cubic spline with 3 degrees of freedom, gender (male vs female), highest education level (compulsory, high school, university), alcohol intake (never, once/month or sometimes, 2-4 times/month, 2-3 times/week, ≥4 times/week), smoking (previous non-regular smoker, non-regular smoker, cigarette smoker, cigar or pipe smoker), occupation (gainfully employed, unemployed, not gainfully employed, retired), and registry data on area level mean income. In the basic model we adjusted only for gender and exposure year. All analyses were performed using R version 3.4.2, and the statistical inference was conducted with a 5% significance level.

3 Results

The mean age at recruitment to this cohort was 45.8 years. Among the 34,748 participants 1,148 cases of IHD were identified during a mean follow-up time of 12.4 years. Table 1 summarizes characteristics of participants according to different levels of leisure time physical activity. Participants not reporting any leisure time exercise were older and more likely to be male, non-commuters and to belong to a lower socioeconomic group. Subjects performing moderate to high-level physical activity, were more likely to be women, non-smokers, and active commuters. The 5-year mean of PM_{10} and $PM_{2.5}$ concentration were different between leisure time physical activity categories, with at most 7 and 6% difference respectively for PM_{10} and $PM_{2.5}$.

For peer review only

Table 1: Characteristics of participants at different levels of exercise in training clothes at baseline

Characteristics	Never	At most once a week	Twice per week or more	p
Exercise in training clothes n (%)	13043 (37.5)	14994 (43.2)	6711 (19.3)	
PM_{10}, µg/m^3 (mean (SD))	10.93 (2.00)	11.06 (1.96)	10.39 (1.94)	<0.001
$PM_{2.5,}$ µg/m^3 (mean (SD))	6.41 (1.12)	6.49 (1.12)	6.11 (1.11)	<0.001
Frequency of active commuting (%)				<0.001
Non-active commuting	6369 (48.8)	6409 (42.7)	2666 (39.7)	
At most two seasons of four	1806 (13.8)	2547 (17.0)	1037 (15.5)	
More than two seasons of four	3506 (26.9)	4976 (33.2)	2555 (38.1)	
Missing	1362 (10.4)	1062 (7.1)	453 (6.8)	
Alcohol intake (%)				<0.001
Never	135 (1.0)	120 (0.8)	79 (1.2)	
Once/month or sometimes	5337 (40.9)	5950 (39.7)	2790 (41.6)	
2-4 times/month	2000 (15.3)	2436 (16.2)	1061 (15.8)	
2-3 times/week	97 (0.7)	93 (0.6)	54 (0.8)	
≥ 4 times/week	5068 (38.9)	5861 (39.1)	2265 (33.8)	
Missing	406 (3.1)	534 (3.6)	462 (6.9)	
Smoking (%)				<0.001
Never smoker	5139 (39.4)	7240 (48.3)	3467 (51.7)	
Previous non-regular smoker	1072 (8.2)	1458 (9.7)	711 (10.6)	
Non-regular smoker	581 (4.5)	807 (5.4)	348 (5.2)	
Previous regular smoker	2729 (20.9)	2865 (19.1)	1201 (17.9)	
Cigarette smoker	2789 (21.4)	1805 (12.0)	426 (6.3)	
Cigar or pipe smoker	186 (1.4)	130 (0.9)	45 (0.7)	
Missing	547 (4.2)	689 (4.6)	513 (7.6)	
Highest education level (%)				<0.001
Compulsory	5534 (42.4)	4319 (28.8)	1369 (20.4)	
High	3573 (27.4)	4116 (27.5)	1951 (29.1)	
University	3449 (26.4)	5933 (39.6)	2911 (43.4)	
Missing	487 (3.7)	626 (4.2)	480 (7.2)	
Gender %, (men)	7072 (54.2)	8036 (53.6)	3274 (48.8)	<0.001
Age, y (mean (SD))	47.28 (9.09)	45.27 (9.02)	44.32 (8.61)	<0.001
Occupation (%)	<0.001			
Gainfully employed	10536 (80.8)	12690 (84.6)	5452 (81.2)	
Unemployed	478 (3.7)	382 (2.5)	174 (2.6)	

Characteristics	Never	At most once a week	Twice per week or more	p
Not gainfully employed	318 (2.4)	352 (2.3)	160 (2.4)	
Retired	673 (5.2)	449 (3.0)	194 (2.9)	
Missing	1038 (8.0)	1121 (7.5)	731 (10.9)	
Mean income for the neighbourhood (SEK)	128285.70 (23017.70)	130332.22 (23875.04)	130221.55 (24605.92)	<0.001

Compared with individuals who reported no exercise, those participants that exercised at least twice per week had a 24% lower risk of IHD (Table 2). The corresponding overall estimate associated with active commuting was a 13% reduced risk of IHD among individuals commuting more than two seasons per year.

Allowing for an interaction between the frequency of exercise in training clothes and particle concentrations (PM_{10} and $PM_{2.5}$) at the home address, the average 24% risk reduction from exercising at least twice per week was found to be driven by an interaction between exercise and particle exposure with 45% and 40% risk reduction among individuals with high PM_{10} and $PM_{2.5}$ concentrations, respectively (table 2). The interaction coefficients estimating the additional benefit of exercise among individuals with a high exposure to particles at their home address were a 3% increased and a 44% reduced risk among those with high PM_{10} concentrations and 0% and 36% risk reductions among those with high $PM_{2.5}$ concentrations.

For active commuters with low particle exposure at their home address, the risk of incident IHD was 17% and 18% higher among those commuting one or two seasons per year and 7% and 3% lower among those commuting at least two seasons per year, for PM_{10} and $PM_{2.5}$ respectively. The benefit of active commuting was larger among individuals with a high particle concentration at their home address: risk reductions for active commuting during one or two seasons were 12% and 13% and for more than two seasons 18% and 21% respectively, compared with non-active commuters. No statistically significant interaction was found between active commuting and particle concentrations at the home address.

For peer review only

Table 2: Hazard ratios (95% CI) for IHD associated with different exercise and commuting habits among persons with different air pollution exposure at home addresses.

Exercise in training clothes	Overall model with no interaction effects	Adjusted[a] HRs in categories of high and low particle exposure		Adjusted[a] interaction hazard ratio
		Low PM_{10}[b]	High PM_{10}[b]	Benefits of exercise/commuting comparing high and low particle exposure
Never	1	1	1	
≤ once/week	1.03 (0.90-1.16)	1.01 (0.84-1.21)	1.04 (0.88-1.23)	1.03 (0.81-1.32)
≥ twice/week	0.76 (0.62-0.93)	0.99 (0.76-1.29)	0.55 (0.40-0.76)	0.56 (0.37-0.84)
		Low $PM_{2.5}$[c]	High $PM_{2.5}$[c]	
Never		1	1	
≤ once/week		1.03 (0.86-1.23)	1.03 (0.87-1.22)	1.00 (0.78-1.28)
≥ twice/week		0.94 (0.72-1.22)	0.60 (0.44-0.82)	0.64 (0.43-0.96)
Active commuting per season		Low PM_{10}[b]	High PM_{10}[b]	
Non-active commuting	1	1	1	
≤ two season of four	1.01 (0.86-1.19)	1.17 (0.93-1.47)	0.88 (0.70-1.11)	0.76 (0.55-1.04)
> two seasons of four	0.87 (0.76-0.998)	0.93 (0.76-1.14)	0.82 (0.68-0.98)	0.88 (0.67-1.15)
		Low $PM_{2.5}$[c]	High $PM_{2.5}$[c]	
Non-active commuting		1	1	
≤ two season of four		1.18 (0.94-1.49)	0.87 (0.69-1.10)	0.73 (0.53-1.01)
> two seasons of four		0.97 (0.80-1.19)	0.79 (0.65-0.95)	0.81 (0.62-1.06)

[a]Adjusted for sex, exposure year, education, smoking, alcohol intake, occupation, mean income, leisure time physical activity, and active commuting

[b]Low PM_{10}: ≤9.6 μg/m^3; High PM_{10}: >9.6 μg/m^3

[c]Low $PM_{2.5}$ ≤5.7 μg/m^3; High $PM_{2.5}$: >5.7 μg/m^3

Individuals exposed to high concentrations of PM_{10} and $PM_{2.5}$ at their home address had a 14% and 1% increased risk of incident IHD respectively, compared to individuals with low concentrations (Table 3). These increased risks were, however, not statistically significant.

For peer review only

When including an interaction between particle concentrations and exercise, risk estimates showed a positive association between air pollution and IHD among individuals performing no exercise and those exercising no more than once a week, whereas a negative association was found among those exercising more than twice a week (Table 3). For PM_{10} concentrations, an increased risk of 21% and 25% was observed among those who did not exercise or exercised at most once a week, respectively, whereas a decreased risk of 32% was estimated among those that exercised at least twice a week. Similarly, for $PM_{2.5}$, the risk of IHD increased by 16% among both those who did not exercise and those who exercised at most once a week, and decreased by 26% among those who exercised at least twice a week. The air pollution-associated risk was lower among those who exercised at least twice a week.

For active commuting, the highest air pollution-associated risk was found among those who did not perform any such activity, among whom the risks increased by a statistically significantly 26% and 24%, respectively (table 3). The risks were, however, not statistically significantly different from air pollution-associated risks among the active commuters (as presented in table 2).

Compared with individuals with low residential particle concentration, a high concentration of PM_{10} was associated with 21% and 25% increased risk for IHD for those who never exercised in training clothes and those who exercised at most once a week, respectively, and showed a 32% decreased risk for those exercising at least twice a week; only the association for those exercising at most once a week was statistically significant. The corresponding estimates associated with $PM_{2.5}$ are increased risk of IHD of 16% for those never exercising and those who exercised at most once a week, and a decreased risk of 26% among those exercising at least twice a week (Table 3); none of these associations was statistically significant.

IHD risk associated with high residential PM_{10} and $PM_{2.5}$ compared with low residential particle concentration was found to be 26% and 24% higher, respectively, among those never actively commuting; both these results were statistically significant. Among those actively commuting one or two seasons per year, IHD risk was 5% and 9% lower, respectively, while among those actively commuting more than two seasons out of four, the risk was 10% and 1% higher, respectively; none of these associations was significant. Overall, no statistically significant modifying effect of exercise and active commuting on the association between high particle concentration at home addresses and IHD was observed (Table3).

Table 3: Hazard ratios (95% CI) for IHD associated with high air pollution levels (vs low) at home address among persons with different exercise/commuting habits.

	Overall model with No interaction effects	Adjusted[a] HRs in different exercise categories		
		Exercise in training clothes		
		Never	≤ once/week	≥ twice/week
Low PM_{10}[b]	1	1	1	1
High PM_{10}[b]	1.14 (0.9-1.45)	1.21 (0.97-1.49)	1.25 (1.01-1.54)	0.68 (0.46-0.998)
Low $PM_{2.5}$[c]	1	1	1	1
High $PM_{2.5}$[c]	1.01 (0.8-1.28)	1.16 (0.94-1.44)	1.16 (0.95-1.43)	0.74 (0.51-1.09)
		Active commuting		
		Non-active commuting	≤ two season of four	> two seasons of four
Low PM_{10}[c]		1	1	1
High PM_{10}[c]		1.26 (1.03-1.54)	0.95 (0.7-1.29)	1.10 (0.86-1.41)
Low $PM_{2.5}$[d]		1	1	1
High $PM_{2.5}$[d]		1.24 (1.02-1.51)	0.91 (0.67-1.24)	1.01 (0.79-1.29)

[a]Adjusted for sex, exposure year, education, smoking, alcohol intake, occupation, mean income, leisure time physical activity, active commuting
[b]Low PM_{10}: ≤9.6 μg/m^3; High PM_{10}: >9.6 μg/m^3

For peer review only

[c]Low $PM_{2.5}$ ≤5.7 µg/m^3; High $PM_{2.5}$: >5.7 µg/m^3

4 Discussion

Overall, we found increased risk of first incident IHD associated with air pollution at the home address but a protective effect of physical activity. A statistically significant beneficial effect of exercise was found among individuals with both high PM_{10} and $PM_{2.5}$, but not among individuals with low levels. Also, for active commuting the benefits were greater among individuals with high residential particle concentrations, but these differences were not statistically significant. Air pollution concentration-associated risks were found among individuals who exercise at most once a week but not among individuals exercising at least twice a week. Statistically significantly increased risks were also found among non-active commuters. These risks were, however, not statistically significantly different from air pollution-associated risks among the active commuters.

Our findings are in accordance with a longitudinal cohort study on MI within the Danish Diet, Cancer, and Health cohort which found an increased benefit of participation in sports among individuals with high NO_2 concentration at the home addresses [16]. The reported risk reduction was 9, 15, and 24% respectively among individuals with low (<14.3 µg/m3), medium (14.3-21 µg/ m3) and high (>21 µg/ m3) residential NO_2 concentration. For walking and cycling they also estimated greater risk reductions for first incident MI among individuals with higher air pollution concentrations. In the same Danish cohort, the long term benefits of physical activity on CVD mortality were also found to be greater among individuals with high residential NO_2 [23]. The risk reduction associated with participation in cycling and gardening among individuals exposed to high residential NO_2 (≥ 19 µg/m3) was greater than those exposed to moderate/low NO_2 concentration (<19 µg/m3). Among participants exposed to high NO_2, the risk reduction for cycling and gardening was 30% and 23%, respectively, whereas among participants exposed to low NO_2, risk reduction was 17% and 15%, respectively. However, the interaction effects in these two studies were not statistically significant.

Opposite findings were observed for the modifying effect of physical activity on the association between air pollution and the incidence of diabetes [15]. Among the participants in the Danish cohort, the risk of developing diabetes increased by 10% per interquartile range of 4.9 mg/m^3 residential NO_2 among physically active individuals, but there was no difference among less physically active individuals. The authors considered that this may be due to an additive rather multiplicative interaction, thus resulting in an increased risk estimate only among physical active individuals with a low risk of developing diabetes.

As inflammation is a causative mechanism for cardiovascular disease, we hypothesized that the anti-inflammatory effects of physical activity may reduce air pollution-associated risks since inflammation is one among several different pathways for the harmful health effects of air pollution. The findings of

For peer review only

greater benefits of physical activity among individuals with higher air pollution exposure for incident IHD risk in our study and incident MI and CVD mortality in the Danish cohort, support such a hypothesis. However, a study on physical activity and white blood cell counts conducted in a large cohort of Taiwanese adults suggested no effect modification by residential air pollution measured as $PM_{2.5}$ [17]. Both physical activity and residential air pollution were, however, found to be associated with an inflammatory response assessed by white blood cell counts. However, the association between physical activity and white blood cell count is variable because exercise also causes a transient increase in white blood cell count which usually normalises within 24 hours [24]. The results of our study cannot be directly compared with the above mentioned studies due to the difference in pollutants [15,16,23] and health outcomes [15,16,23]. This study contributes with air pollution effect estimates on IHD incidence in a population with a relatively low level of air pollution concentrations. Compared with the previous cohort studies on interaction effects between air pollution and physical activity, the annual mean $PM_{2.5}$ concentration was 3-4 times lower. The annual mean in the Taiwanese cohort was 27 µg/m3 and a recent study within the same population as the DHC-cohort studies reported 18 µg/m3. Even at these lower levels of air pollution an increased risk associated with air pollution exposure was found, however not among those who exercised at least twice a week.
A major strength of our study is the air pollution particle concentration exposure data since particles are considered to be the causal component of air pollution [25]. The study used individual time varying exposures of annual mean concentrations during follow-up based on population address registries. The dispersion model used for modelling of particle concentrations has previously been validated [19]. Within the DHC studies NO_2 was used as a proxy for traffic generated air pollution and was assessed only at residential addresses at the year of recruitment or as an annual mean during follow-up. A limitation of our exposure data is that the statistical power only allowed for two exposure categories and therefore no exposure-response assessment was performed.

Another strength of our study is the prospective design, the long follow-up period, the large cohort size and the availability of baseline data on several important confounders. A limitation is the lack of information on the intensity and duration of physical activity and therefore only frequency of exercise could be considered. The study also lacked information on changes in physical activity and other life-style factors during follow-up.

There is a risk of reverse causation if individuals at their baseline examination had a low physical activity level due to poorer health. Furthermore, we lacked information on whether exercise is taking place outdoors or indoors. For active commuters we also lack air pollution exposure calculations during the commute. This would cause exposure misclassification among active commuters with a higher in-traffic air pollution exposure dose compared with non-active commuters, causing a possible bias to the null. This would also occur if individuals chose not to exercise outside during times with high air pollution exposure.

5 Conclusion

The study estimated that exercise reduced the risk of first incident IHD, but only among individuals with higher residential particle concentrations (above median). Similarly, the harmful air pollution effect on IHD was only found among those who exercised less. Our results reinforce the public health message that physical activity is beneficial for cardiovascular health and thus support the adoption of strategies to improve health through promotion of physical activity and mitigation of air pollution. Further studies

For peer review only

are needed to build on the evidence of physical activity and air pollution interactions on the incidence cardiovascular disease. Air pollution exposures during commuting should also be considered in these studies.

Author's contributions: All the authors contributed to the study conception and design. W.R. and J.N.S. analysed the data. W.R wrote the first draft of the manuscript. All authors critically revised the manuscript for important intellectual content and read and approved the final version of the manuscript.

Funding: The study was funded by FORTE (Forskingsrådet för hälsa, arbetsliv och välfärd) [grant number 2012-1296], a Swedish state agency that finances scientific research in health, welfare, and working life, and also by grants from the Swedish state under the agreement between the Swedish government and the county councils (ALF) awarded B F.

Competing interests: None.

Participants consent: Obtained.

Ethics approval: The study was approved by the Regional Ethics Review Board at Umea University (DNR: 2014-136-32M and 2015/16-31Ö).

For peer review only

6 References

1 Lelieveld J, Klingmüller K, Pozzer A, *et al.* Cardiovascular disease burden from ambient air pollution in Europe reassessed using novel hazard ratio functions. *Eur Heart J* Published Online First: 2019. doi:10.1093/eurheartj/ehz135

2 Shrivastava AK, Singh HV, Raizada A, *et al.* C-reactive protein, inflammation and coronary heart disease. Egypt. Hear. J. 2015. doi:10.1016/j.ehj.2014.11.005

3 Cohen AJ, Brauer M, Burnett R, *et al.* Estimates and 25-year trends of the global burden of disease attributable to ambient air pollution: an analysis of data from the Global Burden of Diseases Study 2015. *Lancet* Published Online First: 2017. doi:10.1016/S0140-6736(17)30505-6

4 Münzel T, Gori T, Al-Kindi S, *et al.* Effects of gaseous and solid constituents of air pollution on endothelial function. Eur. Heart J. 2018. doi:10.1093/eurheartj/ehy481

5 Brook RD, Rajagopalan S, Pope CA, *et al.* Particulate matter air pollution and cardiovascular disease: An update to the scientific statement from the American Heart Association. *Circulation* Published Online First: 2010. doi:10.1161/CIR.0b013e3181dbece1

6 Stevens G. Global Health Risks: Mortality and burden of disease attributable to selected major risks. World Heal. Organ. 2009;**87**:646–646. doi:10.2471/BLT.09.070565

7 Lee I, Shiroma EJ, Lobelo F, *et al.* Impact of Physical Inactivity on the World's Major Non-Communicable Diseases. *Lancet* Published Online First: 2012. doi:10.1016/S0140-6736(12)61031-9

8 Nimmo MA, Leggate M, Viana JL, *et al.* The effect of physical activity on mediators of inflammation. Diabetes, Obes. Metab. 2013. doi:10.1111/dom.12156

9 Petersen AMW, Pedersen BK. The anti-inflammatory effect of exercise. *J Appl Physiol* Published Online First: 2005. doi:10.1152/japplphysiol.00164.2004

10 Rajé F, Tight M, Pope FD. Traffic pollution: A search for solutions for a city like Nairobi. *Cities* Published Online First: 2018. doi:10.1016/j.cities.2018.05.008

11 Rojas-Rueda D, De Nazelle A, Teixidó O, *et al.* Health impact assessment of increasing public transport and cycling use in Barcelona: a morbidity and burden of disease approach. *Prev Med (Baltim)* 2013;**57**:573–9.

12 Maizlish N, Woodcock J, Co S, *et al.* Health cobenefits and transportation-related reductions in greenhouse gas emissions in the San Francisco Bay area. *Am J Public Health* 2013;**103**:703–9.

13 Tainio M, de Nazelle AJ, Götschi T, *et al.* Can air pollution negate the health benefits of cycling and walking? *Prev Med (Baltim)* Published Online First: 2015. doi:10.1016/j.ypmed.2016.02.002

14 Giles L V., Koehle MS. The health effects of exercising in air pollution. Sport. Med. 2014. doi:10.1007/s40279-013-0108-z

For peer review only

542 15 Andersen ZJ, Raaschou-Nielsen O, Ketzel M, *et al.* Diabetes incidence and long-term exposure to air pollution: A cohort study. *Diabetes Care* Published Online First: 2012. doi:10.2337/dc11-1155

544 16 Kubesch NJ, Jørgensen JT, Hoffmann B, *et al.* Effects of leisure-time and transport-related physical activities on the risk of incident and recurrent myocardial infarction and interaction with traffic-related air pollution: A cohort study. *J Am Heart Assoc* Published Online First: 2018. doi:10.1161/JAHA.118.009554

548 17 Zhang Z, Hoek G, Chang L yun, *et al.* Particulate matter air pollution, physical activity and systemic inflammation in Taiwanese adults. *Int J Hyg Environ Health* Published Online First: 2018. doi:10.1016/j.ijheh.2017.10.001

551 18 Norberg M, Wall S, Boman K, *et al.* The Västerbotten Intervention Programme: background, design and implications. *Glob Health Action* Published Online First: 2010. doi:10.3402/gha.v3i0.4643

554 19 Segersson D, Eneroth K, Gidhagen L, *et al.* Health impact of PM10, PM2.5 and black carbon exposure due to different source sectors in Stockholm, Gothenburg and Umea, Sweden. *Int J Environ Res Public Health* Published Online First: 2017. doi:10.3390/ijerph14070742

557 20 Hausberger S, Rexeis M, Zallinger M, *et al.* Emission Factors from the Model PHEM for the HBEFA Version 3. 2009.

559 21 Omstedt G, Bringfelt B, Johansson C. A model for vehicle-induced non-tailpipe emissions of particles along Swedish roads. *Atmos Environ* Published Online First: 2005. doi:10.1016/j.atmosenv.2005.06.037

562 22 SMHI. Airviro technical specification, appendix E. 2010. http://www.smhi.se/airviro/2.867/specification/

564 23 Andersen ZJ, de Nazelle A, Mendez MA, *et al.* A study of the combined effects of physical activity and air pollution on mortality in elderly urban residents: The Danish Diet, Cancer, and Health Cohort. *Environ Health Perspect* 2015.

567 24 Abramson N, Melton B. Leukocytosis: Basics of clinical assessment. Am. Fam. Physician. 2000.

568 25 World Health Organization. Review of evidence on health aspects of air pollution – REVIHAAP Project: Technical Report. 2013. doi:10.1007/BF00379640

570

571

For peer review only

STROBE 2007 (v4) Statement—Checklist of items that should be included in reports of *cohort studies*

Section/Topic	Item #	Recommendation	Reported on page #
**Title and abstract**	1	(*a*) Indicate the study's design with a commonly used term in the title or the abstract	1
		(*b*) Provide in the abstract an informative and balanced summary of what was done and what was found	2
**Introduction**			
Background/rationale	2	Explain the scientific background and rationale for the investigation being reported	4
Objectives	3	State specific objectives, including any prespecified hypotheses	4
**Methods**			
Study design	4	Present key elements of study design early in the paper	5
Setting	5	Describe the setting, locations, and relevant dates, including periods of recruitment, exposure, follow-up, and data collection	5
Participants	6	(*a*) Give the eligibility criteria, and the sources and methods of selection of participants. Describe methods of follow-up	5
		(*b*) For matched studies, give matching criteria and number of exposed and unexposed	
Variables	7	Clearly define all outcomes, exposures, predictors, potential confounders, and effect modifiers. Give diagnostic criteria, if applicable	5-6
Data sources/ measurement	8*	For each variable of interest, give sources of data and details of methods of assessment (measurement). Describe comparability of assessment methods if there is more than one group	5-6
Bias	9	Describe any efforts to address potential sources of bias	6
Study size	10	Explain how the study size was arrived at	5
Quantitative variables	11	Explain how quantitative variables were handled in the analyses. If applicable, describe which groupings were chosen and why	5-6
Statistical methods	12	(*a*) Describe all statistical methods, including those used to control for confounding	7
		(*b*) Describe any methods used to examine subgroups and interactions	7
		(*c*) Explain how missing data were addressed	5
		(*d*) If applicable, explain how loss to follow-up was addressed	7
		(*e*) Describe any sensitivity analyses	
**Results**			

For peer review only

Participants	13*	(a) Report numbers of individuals at each stage of study—eg numbers potentially eligible, examined for eligibility, confirmed eligible, included in the study, completing follow-up, and analysed	5
		(b) Give reasons for non-participation at each stage	5
		(c) Consider use of a flow diagram	
Descriptive data	14*	(a) Give characteristics of study participants (eg demographic, clinical, social) and information on exposures and potential confounders	7
		(b) Indicate number of participants with missing data for each variable of interest	8
		(c) Summarise follow-up time (eg, average and total amount)	7
Outcome data	15*	Report numbers of outcome events or summary measures over time	7
Main results	16	(*a*) Give unadjusted estimates and, if applicable, confounder-adjusted estimates and their precision (eg, 95% confidence interval). Make clear which confounders were adjusted for and why they were included	8-11
		(*b*) Report category boundaries when continuous variables were categorized	9, 11
		(*c*) If relevant, consider translating estimates of relative risk into absolute risk for a meaningful time period	
Other analyses	17	Report other analyses done—eg analyses of subgroups and interactions, and sensitivity analyses	9-11
**Discussion**			
Key results	18	Summarise key results with reference to study objectives	12
**Limitations**			
Interpretation	20	Give a cautious overall interpretation of results considering objectives, limitations, multiplicity of analyses, results from similar studies, and other relevant evidence	12-13
Generalisability	21	Discuss the generalisability (external validity) of the study results	13
**Other information**			
Funding	22	Give the source of funding and the role of the funders for the present study and, if applicable, for the original study on which the present article is based	14

*Give information separately for cases and controls in case-control studies and, if applicable, for exposed and unexposed groups in cohort and cross-sectional studies.

Note: An Explanation and Elaboration article discusses each checklist item and gives methodological background and published examples of transparent reporting. The STROBE checklist is best used in conjunction with this article (freely available on the Web sites of PLoS Medicine at http://www.plosmedicine.org/, Annals of Internal Medicine at http://www.annals.org/, and Epidemiology at http://www.epidem.com/). Information on the STROBE Initiative is available at www.strobe-statement.org.

For peer review only

BMJ Open

Air pollution, physical activity and ischemic heart disease - A prospective cohort study of interaction effects

Journal:	*BMJ Open*
Manuscript ID	bmjopen-2020-040912.R1
Article Type:	Original research
Date Submitted by the Author:	04-Jan-2021

Complete List of Authors:	Raza, Wasif; Umeå Universitet Medicinska fakulteten, Public Health and Clinical Medicine, Sustainable Health<br>Krachler, Benno; Umeå Universitet Medicinska fakulteten, Public Health and Clinical Medicine, Sustainable Health<br>Forsberg, Bertil; Umeå Universitet Medicinska fakulteten, Public Health and Clinical Medicine, Sustainable health<br>Sommar, Johan; Umeå Universitet Medicinska fakulteten, Public Health and Clinical Medicine, Sustainable health

**Primary Subject Heading**:	Public health
Secondary Subject Heading:	Epidemiology
Keywords:	Ischaemic heart disease < CARDIOLOGY, PUBLIC HEALTH, Coronary heart disease < CARDIOLOGY

SCHOLARONE™
Manuscripts

I, the Submitting Author has the right to grant and does grant on behalf of all authors of the Work (as defined in the below author licence), an exclusive licence and/or a non-exclusive licence for contributions from authors who are: i) UK Crown employees; ii) where BMJ has agreed a CC-BY licence shall apply, and/or iii) in accordance with the terms applicable for US Federal Government officers or employees acting as part of their official duties; on a worldwide, perpetual, irrevocable, royalty-free basis to BMJ Publishing Group Ltd ("BMJ") its licensees and where the relevant Journal is co-owned by BMJ to the co-owners of the Journal, to publish the Work in this journal and any other BMJ products and to exploit all rights, as set out in our licence.

The Submitting Author accepts and understands that any supply made under these terms is made by BMJ to the Submitting Author unless you are acting as an employee on behalf of your employer or a postgraduate student of an affiliated institution which is paying any applicable article publishing charge ("APC") for Open Access articles. Where the Submitting Author wishes to make the Work available on an Open Access basis (and intends to pay the relevant APC), the terms of reuse of such Open Access shall be governed by a Creative Commons licence – details of these licences and which Creative Commons licence will apply to this Work are set out in our licence referred to above.

Other than as permitted in any relevant BMJ Author's Self Archiving Policies, I confirm this Work has not been accepted for publication elsewhere, is not being considered for publication elsewhere and does not duplicate material already published. I confirm all authors consent to publication of this Work and authorise the granting of this licence.

For peer review only

Air pollution, physical activity and ischemic heart disease - A prospective cohort study of interaction effects

Wasif Raza [1,*], Benno Krachler [1], Bertil Forsberg [1] and Johan Nilsson Sommar [1]

[1] Department of Public Health and Clinical Medicine, Section of Sustainable Health, Umeå University, Sweden; wasif.raza@umu.se (W.R.); benno.krachler@umu.se (B.K.); bertil.forsberg@umu.se (B.F.); johan.sommar@umu.se (J.N.S.)

* Correspondence: wasif.raza@umu.se ; Tel.: +46-72-289-5144

For peer review only

Abstract

Objective: To assess a possible interaction effect between physical activity and air pollution on first incidence of ischemic heart disease (IHD).

Design: Prospective cohort study

Setting: Umeå, Northern Sweden

Participants: We studied 34,748 adult participants of Västerbotten Intervention Programme cohort from 1990 to January 2014. Annual particulate matter concentrations ($PM_{2.5}$ and PM_{10}) at the participants' residential addresses were modelled and a questionnaire on frequency of exercise and active commuting was completed at baseline. Cox proportional hazards modelling was used to estimate 1) association with physical activity at different levels of air pollution, and 2) the association with particulate matter at different levels of physical activity.

Outcome: First incidence of IHD.

Results: Over a mean follow-up of 12.4 years, there were 1,148 IHD cases. Overall, we observed an increased risk of IHD among individuals with higher concentrations of particles at their home address. Exercise at least twice a week was associated with a lower risk of IHD among participants with high residential $PM_{2.5}$ (0.60; 95% CI: 0.44-0.82) and PM_{10} (0.55; 95% CI: 0.4-0.76). The same beneficial effect was not observed with low residential $PM_{2.5}$ (0.94; 95% CI: 0.72-1.22) and PM_{10} (0.99; 95% CI: 0.76-1.29). An increased risk associated with higher long-term exposure to particles was only observed among participants never exercising in training clothes and among those not performing any active commuting. However, only the interaction effect on hazard ratios for exercise was statistically significant.

Conclusion: Exercise was associated with a lower risk of first incidence of IHD among individuals with higher residential particle concentrations. An air pollution-associated risk was only observed among those who exercised less. The findings support the promotion of physical activity and a mitigation of air pollution.

Key Words: Air pollution, $PM_{2.5}$, PM_{10}, exercise, interaction, ischemic heart disease

For peer review only

Strengths and limitations of this study

- This study simultaneously evaluated the impact of physical activity and air pollution and their interaction on first incident IHD cases in a population with relatively low level of air pollution concentrations.
- For air pollution exposure, this study used individual time varying exposures of annual mean concentrations during follow-up based on population address registries.
- Another strength is the prospective design and the availability of baseline data on several important confounders.
- No exposure-response assessment could be performed since the statistical power only allowed for the formation of two exposure categories.
- Differences in air pollution exposure during active commuting might cause biased estimates due to exposure misclassification.

For peer review only

1 Introduction

Cardiovascular disease (CVD) is the most important cause of morbidity and premature mortality worldwide, accounting for 422.3 million cases and 17.92 million deaths in 2015 [1]. There is solid evidence that inflammation is a key upstream pathogenic mechanism [2]. Ambient air pollution, generally comprising ozone, sulphur dioxide, nitrogen dioxide and particulate matter (PM), is a leading contributor to the global burden of disease and an important risk factor for morbidity and mortality. Particulate matter is often measured as $PM_{2.5}$, to represent particles with a diameter of 2.5 micrometres or less, and PM_{10}, to represent particles with a diameter of 10 micrometres or less. Ambient $PM_{2.5}$ exposure alone has been estimated to account for 4.2 million deaths in 2015, of which 1.5 million deaths were caused by ischemic heart diseases (IHD) [3]. The underlying mechanisms mediating the pathogenic impact of air pollution involve systemic oxidative stress and inflammation [4,5]. Physical activity is a salutogenic factor in numerous non-communicable chronic diseases (NCDs), with physical inactivity being responsible for 6% of the burden of disease from coronary heart disease [6,7]. The beneficial effects of physical activity include protection against low-grade inflammation by releasing anti-inflammatory substances, such as interleukin 6, from contracting muscles [8,9].

Rapid urbanization and increased use of motorized transport contribute to modern day problems such as traffic congestion, traffic related air pollution and lack of physical activity. Promotion of active transportation by changing mode of transport from car to cycling and walking are among the different strategies used to tackle these challenges [10–12]. As inflammation is a causative mechanism for cardiovascular disease, it is conceivable that the anti-inflammatory effects of physical activity may mitigate the harmful effects associated with exposure to air pollution. However, one major concern with physical activity in a polluted environment is the increased inhalation of particles due to an increase in respiratory volume that may counteract the beneficial effects of physical activity [13,14].

The long-term effects of air pollution among individuals with different levels of leisure time physical activity have been estimated within the Danish Diet, Cancer, and Health cohort. The incidence of diabetes was assessed in relation to leisure time physical activity and nitrogen dioxide (NO_2) concentration at the home address. Residential NO_2 was found to be associated with increased incidence of diabetes, but only among physically active individuals [15]. As far as we know, only one study has examined the modifying effect of air pollution on the association between physical activity and cardiovascular disease. In their recent study, Kubesch and colleagues conclude that physical activity reduced the risk of first incidence of myocardial infarction (MI) and recurrent MI among individuals with high NO_2 concentration at the residential addresses [16]. As only one pollutant (NO_2) and only one cardiovascular outcome (MI) have been studied, the knowledge of a possible interaction between air pollution and physical activity on CVD is inconclusive.

A Taiwanese study found an independent inverse association of habitual physical activity with inflammation across different levels of $PM_{2.5}$ exposure, although long term exposure was associated with increased inflammation at all levels of physical activity [17].

We therefore aimed to examine interaction effects between physical activity and long-term exposure to $PM_{2.5}$ and PM_{10} at residential addresses on the incidence of ischemic heart disease (IHD). We wanted to assess: 1) whether air pollution modifies the beneficial effects of physical activity on IHD, and 2) whether physical activity modifies the harmful effects of air pollution on IHD.

2 Methods

To determine the interaction effect between air pollution and physical activity on IHD incidence, we combined cohort data which comprised risk factors for IHD, national registry data on IHD incidence from the Swedish National Board of Health and Welfare, and yearly annual mean air pollution particle concentrations at the individuals' residential addresses using dispersion models from the Swedish Clean Air and Climate Research Program (SCAC).

VIP (Västerbotten Intervention Programme) is a population based screening and prospective cohort study, developed to reduce the risk of future CVD and diabetes by promoting a healthy lifestyle among individuals living in the Västerbotten region. The SCAC developed methods to estimate exposure to source-specific particulate matter such as $PM_{2.5}$ and PM_{10} and at residential addresses in Gothenborg, Stockholm and Umeå.

2.1 Study population

VIP is an ongoing population-based health investigation survey of all individuals at ages 40, 50 or 60 years, depending upon risk factors, living in the Västerbotten region, who are invited to participate in systematic risk factor screening and individual counselling about healthy lifestyle habits. A detailed description of VIP has been presented elsewhere [18]. Between 1990 and 2014, a total of 42,488 of the VIP participants that lived in Umeå Municipality during the study period were included in the analysis. After exclusion of 7,740 participants with missing information on exercise, the study sample thus consisted of 34,748 individuals, 53% men and 47% women, 40-60 years of age at baseline examination, with no previous history of IHD at time of enrolment. After exclusion of individuals with missing information on included confounders the final number of included individuals were 31424 and 29218 for the analyses of exercise in training clothes and active commuting, respectively. All participants in the VIP gave their informed written consent, and the study was approved by the Regional Ethics Review Board at Umea University (DNR: 2014-136-32M and 2015/16-31Ö).

2.2 Leisure time exercise and active commuting

The VIP questionnaire includes various self-reported information on physical activity including frequency of leisure time physical activity and active commuting (cycling or walking to and from work). The association with IHD was assessed in relation to frequency of exercise in training clothes and amounts of active commuting. Exercise during the previous three months was categorized as never, rarely, once per week, 2-3 times per week or more than 3 times per week. Based on this information, participants were categorized as "Twice per week or more" if they exercised with a frequency of 2-3 times per week and more, "At most once a week" if they exercised rarely or once per week, and "Never" if no activity was performed. For active commuting, participants were asked about their mode of transport to work each season. Participants were classified in three categories: "Non- active commuting" if commuting every season by car or bus, "At most two seasons out of four" if cycling or walking at most half a year and "More than two seasons out of four", if cycling and walking more than half a year.

For peer review only

2.3 Covariates

The VIP questionnaire also gathered information on participants' educational status, occupation, smoking, alcohol intake and economic status. Education was defined according to the International Standard Classification of Education (ISCED), United Nations Educational, Scientific and Cultural Organization (UNESCO) 1997. Participants were asked about the highest level education they had achieved with eight predefined categories ranging from "pre-school" to "university education." Alcohol intake was assessed by the reported frequency of consumption with answering options that ranged from "Never" to "2-4 times/week." Information on smoking was gathered by using the question, "How often do you smoke?" Information on occupational status was obtained with a question, "What kind of job do you have nowadays?" with the answering options of eight predefined categories. Finally, information on occupation status was asked with the question, "What is your current occupation?" with 8 categories ranging from permanently employed to retired.

2.4 Air pollution concentrations

Annual mean total concentrations of $PM_{2.5}$ and PM_{10} for the years 1990-2011 (and thereafter linearly extrapolated up until year 2014) were obtained from the SCAC research program, described in detail elsewhere [19]. Briefly, concentrations of $PM_{2.5}$ and PM_{10} were estimated within SCAC by applying dispersion models on local or regional emission inventories. These emission inventories contain detailed information on emissions from different source categories, such as road traffic exhaust, road traffic non-exhaust, domestic heating, shipping and industrial activities. For Umeå, inventories were validated through monitoring for consistency. Emissions from small-scale residential heating were assessed using registry data from chimney sweepers that included the type of wood stove or boiler. The addresses of these residences were geocoded using the geographical centre coordinate of the estate. Road traffic emission factors for PM exhaust for different vehicle types, speeds and driving conditions were calculated based on the Handbook Emission Factors for Road Transport (HBEFA) version 3.1 [20]. Estimates for non-exhaust contribution from brake and tire wear were based on Omstedt et al. [21]. The annual average emission from shipping was used in the modelling on a 1x1 km^2 grid resolution. The emissions from other sources such as industrial processes, off-road machinery and agriculture was collected from Swedish Meteorological and Hydrological Institute (SMHI). To obtain annual average emissions of $PM_{2.5}$ and PM_{10}, Gaussian models included in the Airviro air quality management system (SMHI, 2010) were used for simulation based on hourly meteorological data for 1990, 2000 and 2011 [22]. The comparison between measured and modelled $PM_{2.5}$ and PM_{10} agreed well at most monitoring stations (r^2=0.87 and r^2=0.65, respectively).

2.5 Outcome

We linked the records in VIP and through the unique Swedish personal identification number with data on first IHD event cases from the National Patient Register and the Cause of Death Register, both at the National Board of Health and Welfare, using primary discharge diagnoses for IHD according to International Classification of Diseases, 10th revision (ICD-10): code I20-125.

For peer review only - http://bmjopen.bmj.com/site/about/guidelines.xhtml

2.6 Patient and public involvement

No patients were involved in this study.

2.7 Statistical Analysis

We performed survival analyses using Cox regression proportion hazard model to estimate hazard ratios (HRs), and 95% confidence intervals (CIs), to estimate 1) the association between first incident IHD and air pollution exposure at different levels of physical activity and 2) the association between first incident IHD and physical activity at different levels of air pollution exposure. Age was used as the underlying timescale since it is a stronger confounder than calendar time. Follow-up started at date of recruitment to the cohort and ended with the earliest of the date of first IHD case, emigration, death, or 31 December 2013. Interaction between physical activity and air pollution and their impact on IHD was studied by introducing an interaction term into the model. Residential annual mean particle concentrations were used to calculate moving averages over the recent five years which were thereafter categorized as below or above the median concentration for $PM_{2.5}$ and PM10, respectively. Interaction with physical activity was assessed based on 1) the frequency of exercise in training clothes and 2) the number of seasons the individual walked or cycled to work. Active commuting by walking or cycling was categorized into three groups: non-active commuters, active commuters at most half of the year (up to two out of four seasons), active commuters more than half a year (more than two out of four seasons). Estimates were adjusted for: calendar year as a penalized cubic spline with 3 degrees of freedom, gender (male vs female), highest education level (compulsory, high school, university), alcohol intake (never, once/month or sometimes, 2-4 times/month, 2-3 times/week, ≥4 times/week), smoking (previous non-regular smoker, non-regular smoker, cigarette smoker, cigar or pipe smoker), occupation (gainfully employed, unemployed, not gainfully employed, retired), and registry data on area level mean income. In the basic model we adjusted only for gender and exposure year. All analyses were performed using R version 3.4.2, and the statistical inference was conducted with a 5% significance level. T-tests, global analysis of variance tests (ANOVA) and chi-square tests were used to test for differences in means and proportions of covariates between categories of exercise in training clothes (Table 1). The Schoenfeld residuals test was used to assess the assumption of proportional hazards. A sensitivity analysis was conducted by excluding participants with short follow-up time.

3 Results

The mean age at recruitment to this cohort was 45.8 years. Among the 34,748 participants 1,148 cases of IHD were identified during a mean follow-up time of 12.4 years. Of those cases, 500 never exercised, 529 exercised at most once a week and 119 exercised at least twice a week. Table 1 summarizes characteristics of participants according to different levels of leisure time physical activity. Participants not reporting any leisure time exercise were older and more likely to be male, non-commuters and to belong to a lower socioeconomic group. Subjects performing moderate to high-level physical activity, were more likely to be women, non-smokers, and active commuters. The 5-year mean of PM_{10} and $PM_{2.5}$ concentration were different between leisure time physical activity categories, with at most 7 and 6% difference respectively for PM_{10} and $PM_{2.5}$.

For peer review only

Table 1: Characteristics of participants at different levels of exercise in training clothes at baseline

Characteristics	Never	At most once a week	Twice per week or more	p
Exercise in training clothes n (%)	13043 (37.5)	14994 (43.2)	6711 (19.3)	
PM_{10}, $\mu g/m^3$ (mean (SD))	10.93 (2.00)	11.06 (1.96)	10.39 (1.94)	<0.001
$PM_{2.5,}$ $\mu g/m^3$ (mean (SD))	6.41 (1.12)	6.49 (1.12)	6.11 (1.11)	<0.001
Frequency of active commuting (%)				<0.001
Non-active commuting	6369 (48.8)	6409 (42.7)	2666 (39.7)	
At most two seasons of four	1806 (13.8)	2547 (17.0)	1037 (15.5)	
More than two seasons of four	3506 (26.9)	4976 (33.2)	2555 (38.1)	
Missing	1362 (10.4)	1062 (7.1)	453 (6.8)	
Alcohol intake (%)				<0.001
Never	135 (1.0)	120 (0.8)	79 (1.2)	
Once/month or sometimes	5337 (40.9)	5950 (39.7)	2790 (41.6)	
2-4 times/month	2000 (15.3)	2436 (16.2)	1061 (15.8)	
2-3 times/week	97 (0.7)	93 (0.6)	54 (0.8)	
≥ 4 times/week	5068 (38.9)	5861 (39.1)	2265 (33.8)	
Missing	406 (3.1)	534 (3.6)	462 (6.9)	
Smoking (%)				<0.001
Never smoker	5139 (39.4)	7240 (48.3)	3467 (51.7)	
Previous non-regular smoker	1072 (8.2)	1458 (9.7)	711 (10.6)	
Non-regular smoker	581 (4.5)	807 (5.4)	348 (5.2)	
Previous regular smoker	2729 (20.9)	2865 (19.1)	1201 (17.9)	
Cigarette smoker	2789 (21.4)	1805 (12.0)	426 (6.3)	
Cigar or pipe smoker	186 (1.4)	130 (0.9)	45 (0.7)	
Missing	547 (4.2)	689 (4.6)	513 (7.6)	
Highest education level (%)				<0.001
Compulsory	5534 (42.4)	4319 (28.8)	1369 (20.4)	
High	3573 (27.4)	4116 (27.5)	1951 (29.1)	
University	3449 (26.4)	5933 (39.6)	2911 (43.4)	
Missing	487 (3.7)	626 (4.2)	480 (7.2)	
Gender %, (men)	7072 (54.2)	8036 (53.6)	3274 (48.8)	<0.001
Age, y (mean (SD))	47.28 (9.09)	45.27 (9.02)	44.32 (8.61)	<0.001
Occupation (%)	<0.001			
Gainfully employed	10536 (80.8)	12690 (84.6)	5452 (81.2)	
Unemployed	478 (3.7)	382 (2.5)	174 (2.6)	
Not gainfully employed	318 (2.4)	352 (2.3)	160 (2.4)	
Retired	673 (5.2)	449 (3.0)	194 (2.9)	
Missing	1038 (8.0)	1121 (7.5)	731 (10.9)	

Characteristics	Never	At most once a week	Twice per week or more	p
Mean income for the neighbourhood (SEK)	128285.70 (23017.70)	130332.22 (23875.04)	130221.55 (24605.92)	<0.001

Compared with individuals who reported no exercise, those participants that exercised at least twice per week had a 24% lower risk of IHD (Table 2). The corresponding overall estimate associated with active commuting was a 13% reduced risk of IHD among individuals commuting more than two seasons per year.

Allowing for an interaction between the frequency of exercise in training clothes and particle concentrations (PM_{10} and $PM_{2.5}$) at the home address, the average 24% risk reduction from exercising at least twice per week was found to be driven by statistically significant interaction between exercise and particle exposure with 45% and 40% risk reduction among individuals with high PM_{10} and $PM_{2.5}$ concentrations, respectively (table 2). The interaction coefficients estimating the additional benefit of exercise among individuals with a high PM_{10} concentration at their home addresses were a 3% increased risk among those who exercised at most once a week whereas a decreased risk of 44% was estimated among those who exercised at least twice a week. The corresponding estimates among those with high $PM_{2.5}$ concentrations were a risk reduction of 0% and 36% respectively.

For active commuters with low particle exposure at their home address, the risk of incident IHD was 17% and 18% higher among those commuting one or two seasons per year and 7% and 3% lower among those commuting at least two seasons per year, for PM_{10} and $PM_{2.5}$ respectively. The benefit of active commuting was larger among individuals with a high particle concentration at their home address: risk reductions for active commuting during one or two seasons were 12% and 13% and for more than two seasons 18% and 21% respectively, compared with non-active commuters. No statistically significant interaction was found between active commuting and particle concentrations at the home address.

For peer review only

Table 2: Hazard ratios (95% CI) for IHD associated with different exercise and commuting habits among persons with different air pollution exposure at home addresses.

Exercise in training clothes	Overall model with no interaction effects	Proportional hazard p[d]-value *	Adjusted[a] HRs in categories of high and low particle exposure				Adjusted[a] interaction hazard ratio	
			Low PM_{10}[b]	Proportional hazard p-value[d]	High PM_{10}[b]	Proportional hazard p-value[d]	Benefits of exercise/commuting comparing high and low particle exposure	Proportional hazard p-value[d]
Never	1		1		1		1.03 (0.81-1.32)	0.75
≤ once/week	1.03 (0.90-1.16)	0.35	1.01 (0.84-1.21)	0.67	1.04 (0.88-1.23)	0.35	0.56 (0.37-0.84)	0.65

≥ twice/week	0.76 (0.62-0.93)	0.08	0.99 (0.76-1.29)	0.47	0.55 (0.40-0.76)	0.24		
			Low $PM_{2.5}$[c]		High $PM_{2.5}$[c]			
Never			1		1		1.00 (0.78-1.28)	0.61
≤ once/week			1.03 (0.86-1.23)	0.78	1.03 (0.87-1.22)	0.30	0.64 (0.43-0.96)	0.30
≥ twice/week			0.94 (0.72-1.22)	0.69	0.60 (0.44-0.82)	0.09		
Active commuting per season			Low PM_{10}[b]		High PM_{10}[b]			
Non-active commuting	1		1		1			
≤ two seasons of four	1.01 (0.86-1.19)	0.50	1.17 (0.93-1.47)	0.35	0.88 (0.70-1.11)	0.70	0.76 (0.55-1.04)	0.34
> two seasons of four	0.87 (0.76-0.998)	0.85	0.93 (0.76-1.14)	0.48	0.82 (0.68-0.98)	0.89	0.88 (0.67-1.15)	0.53
			Low $PM_{2.5}$[c]		High $PM_{2.5}$[c]			
Non-active commuting			1		1			
≤ two seasons of four			1.18 (0.94-1.49)	0.43	0.87 (0.69-1.10)	0.76	0.73 (0.53-1.01)	0.43
> two seasons of four			0.97 (0.80-1.19)	0.37	0.79 (0.65-0.95)	0.89	0.81 (0.62-1.06)	0.44

[a]Adjusted for sex, calendar year, education, smoking, alcohol intake, occupation, neighbourhood mean income, leisure time physical activity, and active commuting

[b]Low PM_{10}: ≤9.6 µg/m^3; High PM_{10}: >9.6 µg/m^3

[c]Low $PM_{2.5}$ ≤5.7 µg/m^3; High $PM_{2.5}$: >5.7 µg/m^3

[d] p-value of the Schoenfeld residual test of proportional hazards

Individuals exposed to high concentrations of PM_{10} and $PM_{2.5}$ at their home address had a 14% and 1% increased risk of incident IHD respectively, compared to individuals with low concentrations (Table 3). These increased risks were, however, not statistically significant.

When including an interaction between particle concentrations and exercise, risk estimates showed a positive association between air pollution and IHD among individuals performing no exercise and those exercising no more than once a week, whereas a negative association was found among those exercising more than twice a week (Table 3).

Compared with individuals with low residential particle concentration, a high concentration of PM_{10} was associated with 21% and 25% increased risk for IHD for those who never exercised in training clothes and those who exercised at most once a week, respectively, and showed a 32% decreased risk for those exercising at least twice a week; only the association for those exercising at most once a week was statistically significant. The corresponding estimates associated with $PM_{2.5}$ are increased risk of IHD of 16% for those never exercising and those who exercised at most once a week, and a decreased risk of 26% among those exercising at least twice a week (Table 3); none of these associations was statistically significant.

For peer review only

IHD risk associated with high residential PM_{10} and $PM_{2.5}$ compared with low residential particle concentration was found to be 26% and 24% higher, respectively, among those never actively commuting; both these results were statistically significant. Among those actively commuting one or two seasons per year, IHD risk was 5% and 9% lower, respectively, while among those actively commuting more than two seasons out of four, the risk was 10% and 1% higher, respectively; none of these risks were, however, statistically significantly different from the air pollution-associated risks among the active commuters (Table 2). Overall, no statistically significant modifying effect of exercise and active commuting on the association between high particle concentration at home addresses and IHD was observed (Table3).

Excluding participants with short follow-up time did not affect the main conclusions of our study, however estimates tended to be lower for overall effect of air pollution on IHD (Supplementary file A: Table 2).

Table 3: Hazard ratios (95% CI) for IHD associated with high air pollution levels (vs low) at home address among persons with different exercise/commuting habits.

	Overall model with no interaction effects	Proportional hazard p-value[d]	Adjusted[a] HRs in categories of high and low particle exposure					
			Exercise in training clothes					
			Never	Proportional hazard p-value[d]	≤ once/week	Proportional hazard p-value[d]	≥ twice/week	Proportional hazard p-value[d]
Low PM_{10}[b]	1		1		1		1	
High PM_{10}[b]	1.14 (0.9-1.45)	0.55	1.21 (0.97-1.49)	0.71	1.25 (1.01-1.54)	0.45	0.68 (0.46-0.998)	0.50
Low $PM_{2.5}$[c]	1		1		1		1	
High $PM_{2.5}$[c]	1.01 (0.8-1.28)	1.10	1.16 (0.94-1.44)	0.38	1.16 (0.95-1.43)	0.13	0.74 (0.51-1.09)	0.11
			Active commuting					
			Non-active commuting		≤ two seasons of four	P-value	> two seasons of four	
Low PM_{10}[b]			1		1		1	
High PM_{10}[b]			1.26 (1.03-1.54)	0.39	0.95 (0.7-1.29)	0.66	1.10 (0.86-1.41)	0.17
Low $PM_{2.5}$[c]			1		1		1	
High $PM_{2.5}$[c]			1.24 (1.02-1.51)	0.11	0.91 (0.67-1.24)	0.84	1.01 (0.79-1.29)	0.04

[a]Adjusted for sex, calendar year, education, smoking, alcohol intake, occupation, neighbourhood mean income, leisure time physical activity, active commuting
[b]Low PM_{10}: ≤9.6 µg/m^3; High PM_{10}: >9.6 µg/m^3
[c]Low $PM_{2.5}$ ≤5.7 µg/m^3; High $PM_{2.5}$: >5.7 µg/m^3
[d] p-value of the Schoenfeld residual test of proportional hazards

For peer review only

4 Discussion

Overall, we found increased risk of first incident IHD associated with air pollution at the home address but a protective effect of physical activity. A statistically significant beneficial effect of exercise was found among individuals with high PM10/PM2.5, but not among individuals with low levels. Also, for active commuting the benefits were greater among individuals with high residential particle concentrations, but these differences were not statistically significant. Air pollution concentration-associated risks were found among individuals who exercise at most once a week but not among individuals exercising at least twice a week. Statistically significantly increased risks were also found among non-active commuters. These risks were, however, not statistically significantly different from air pollution-associated risks among the active commuters.

Our findings are in accordance with a longitudinal cohort study on MI within the Danish Diet, Cancer, and Health cohort which found an increased benefit of participation in sports among individuals with high NO_2 concentration at the home addresses [16]. The reported risk reduction was 9, 15, and 24% respectively among individuals with low (<14.3 µg/m3), medium (14.3-21 µg/ m3) and high (>21 µg/ m3) residential NO_2 concentration. For walking and cycling they also estimated greater risk reductions for first incident MI among individuals with higher air pollution concentrations. In the same Danish cohort, the long term benefits of physical activity on CVD mortality were also found to be greater among individuals with high residential NO_2 [23]. The risk reduction associated with participation in cycling and gardening among individuals exposed to high residential NO_2 (≥ 19 µg/m3) was greater than those exposed to moderate/low NO_2 concentration (<19 µg/m3). Among participants exposed to high NO_2, the risk reduction for cycling and gardening was 30% and 23%, respectively, whereas among participants exposed to low NO_2, risk reduction was 17% and 15%, respectively. However, the interaction effects in these two studies were not statistically significant.

Opposite findings were observed for the modifying effect of physical activity on the association between air pollution and the incidence of diabetes [15]. Among the participants in the Danish cohort, the risk of developing diabetes increased by 10% per interquartile range of 4.9 mg/m^3 residential NO_2 among physically active individuals, but there was no difference among less physically active individuals. The authors considered that this may be due to an additive rather multiplicative interaction, thus resulting in an increased risk estimate only among physical active individuals with a low risk of developing diabetes.

As inflammation is a causative mechanism for cardiovascular disease, we hypothesized that the anti-inflammatory effects of physical activity may reduce air pollution-associated risks since inflammation is one among several different pathways for the harmful health effects of air pollution. The findings of greater benefits of physical activity among individuals with higher air pollution exposure for incident IHD

risk in our study and incident MI and CVD mortality in the Danish cohort, support such a hypothesis. However, a study on physical activity and white blood cell counts conducted in a large cohort of Taiwanese adults suggested no effect modification by residential air pollution measured as $PM_{2.5}$ [17]. Both physical activity and residential air pollution were, however, found to be associated with an inflammatory response assessed by white blood cell counts. However, the association between physical activity and white blood cell count is variable because exercise also causes a transient increase in white blood cell count which usually normalises within 24 hours [24]. The results of our study cannot be directly compared with the above mentioned studies due to the difference in pollutants [15,16,23] and health outcomes [15,16,23]. This study contributes with air pollution effect estimates on IHD incidence in a population with a relatively low level of air pollution concentrations. Compared with the previous cohort studies on interaction effects between air pollution and physical activity, the annual mean $PM_{2.5}$ concentration was 3-4 times lower. The annual mean in the Taiwanese cohort was 27 µg/m3 and a recent study within the same population as the DHC-cohort studies reported 18 µg/m3. Even at these lower levels of air pollution an increased risk associated with air pollution exposure was found, however not among those who exercised at least twice a week. Even though active commuting may result in higher air pollution exposure compared with for instance driving a car to work, the risk of an IHD event was still reduced since the benefit of the physical activity was greater than the IHD risk imposed by the air pollution exposure.

A major strength of our study is the air pollution particle concentration exposure data since particles are considered to be the causal component of air pollution [25]. The study used individual time varying exposures of annual mean concentrations during follow-up based on population address registries. The dispersion model used for modelling of particle concentrations has previously been validated [19]. Within the DHC studies NO_2 was used as a proxy for traffic generated air pollution and was assessed only at residential addresses at the year of recruitment or as an annual mean during follow-up. A limitation of our exposure data is that the statistical power only allowed for two exposure categories and therefore no exposure-response assessment was performed.

Another strength of our study is the prospective design, the long follow-up period, the large cohort size and the availability of baseline data on several important confounders. A limitation is the lack of information on the intensity and duration of physical activity and therefore only frequency of exercise could be considered. The study also lacked information on changes in physical activity and other life-style factors during follow-up as the information was only retrieved at baseline.

There is a risk of reverse causation if individuals at their baseline examination had a low physical activity level due to poorer health. Individuals could for instance have diseases that affect their risk to later in life have an IHD event (such as diabetes) prior to baseline examination. If this prior disease also affected the frequency the individual exercised in training clothes, or mode of commuting, then a reverse causation between physical activity and IHD risk may occur. Individuals with a prior IHD event at baseline were however excluded. Furthermore, we lacked information on whether exercise is taking place outdoors or indoors. For active commuters we also lack air pollution exposure calculations during the commute. This would cause exposure misclassification among active commuters with a higher in-traffic air pollution exposure dose compared with non-active commuters, causing a possible bias to the null. This would also occur if individuals chose not to exercise outside during times with high air pollution exposure.

For peer review only

5 Conclusion

The study estimated that exercise reduced the risk of first incident IHD, but only among individuals with higher residential particle concentrations (above median). Similarly, the harmful air pollution effect on IHD was only found among those who exercised less. Our results reinforce the public health message that physical activity is beneficial for cardiovascular health and thus support the adoption of strategies to improve health through promotion of physical activity and mitigation of air pollution. Further studies are needed to build on the evidence of physical activity and air pollution interactions on the incidence cardiovascular disease. Air pollution exposures during commuting should also be considered in these studies.

Author's contributions: All the authors contributed to the study conception and design. W.R. and J.N.S. analysed the data. W.R wrote the first draft of the manuscript. B.F., B.K. and J.N.S. critically revised the manuscript for important intellectual content. All authors read and approved the final version of the manuscript.

Funding: The study was funded by FORTE (Forskingsrådet för hälsa, arbetsliv och välfärd) [grant number 2012-1296], a Swedish state agency that finances scientific research in health, welfare, and working life, and by grants from Folksam research foundation and the Swedish state under the agreement between the Swedish government and the county councils (ALF) awarded B F [grant number: VLL-645781].

Competing interests: None.

Participants consent: Obtained.

Ethics approval: The study was approved by the Regional Ethics Review Board at Umea University (DNR: 2014-136-32M and 2015/16-31Ö).

Data availability: No additional data is available

For peer review only

6 References

1 Lelieveld J, Klingmüller K, Pozzer A, *et al.* Cardiovascular disease burden from ambient air pollution in Europe reassessed using novel hazard ratio functions. *Eur Heart J* Published Online First: 2019. doi:10.1093/eurheartj/ehz135

2 Shrivastava AK, Singh HV, Raizada A, *et al.* C-reactive protein, inflammation and coronary heart disease. Egypt. Hear. J. 2015. doi:10.1016/j.ehj.2014.11.005

3 Cohen AJ, Brauer M, Burnett R, *et al.* Estimates and 25-year trends of the global burden of disease attributable to ambient air pollution: an analysis of data from the Global Burden of Diseases Study 2015. *Lancet* Published Online First: 2017. doi:10.1016/S0140-6736(17)30505-6

4 Münzel T, Gori T, Al-Kindi S, *et al.* Effects of gaseous and solid constituents of air pollution on endothelial function. Eur. Heart J. 2018. doi:10.1093/eurheartj/ehy481

5 Brook RD, Rajagopalan S, Pope CA, *et al.* Particulate matter air pollution and cardiovascular disease: An update to the scientific statement from the American Heart Association. *Circulation* Published Online First: 2010. doi:10.1161/CIR.0b013e3181dbece1

6 Stevens G. Global Health Risks: Mortality and burden of disease attributable to selected major risks. World Heal. Organ. 2009;**87**:646–646. doi:10.2471/BLT.09.070565

7 Lee I, Shiroma EJ, Lobelo F, *et al.* Impact of Physical Inactivity on the World's Major Non-Communicable Diseases. *Lancet* Published Online First: 2012. doi:10.1016/S0140-6736(12)61031-9

8 Nimmo MA, Leggate M, Viana JL, *et al.* The effect of physical activity on mediators of inflammation. Diabetes, Obes. Metab. 2013. doi:10.1111/dom.12156

9 Petersen AMW, Pedersen BK. The anti-inflammatory effect of exercise. *J Appl Physiol* Published Online First: 2005. doi:10.1152/japplphysiol.00164.2004

10 Rajé F, Tight M, Pope FD. Traffic pollution: A search for solutions for a city like Nairobi. *Cities* Published Online First: 2018. doi:10.1016/j.cities.2018.05.008

11 Rojas-Rueda D, De Nazelle A, Teixidó O, *et al.* Health impact assessment of increasing public transport and cycling use in Barcelona: a morbidity and burden of disease approach. *Prev Med (Baltim)* 2013;**57**:573–9.

12 Maizlish N, Woodcock J, Co S, *et al.* Health cobenefits and transportation-related reductions in greenhouse gas emissions in the San Francisco Bay area. *Am J Public Health* 2013;**103**:703–9.

13 Tainio M, de Nazelle AJ, Götschi T, *et al.* Can air pollution negate the health benefits of cycling and walking? *Prev Med (Baltim)* Published Online First: 2015. doi:10.1016/j.ypmed.2016.02.002

14 Giles L V., Koehle MS. The health effects of exercising in air pollution. Sport. Med. 2014. doi:10.1007/s40279-013-0108-z

15 Andersen ZJ, Raaschou-Nielsen O, Ketzel M, *et al.* Diabetes incidence and long-term exposure to air pollution: A cohort study. *Diabetes Care* Published Online First: 2012. doi:10.2337/dc11-1155

For peer review only

16 Kubesch NJ, Jørgensen JT, Hoffmann B, *et al.* Effects of leisure-time and transport-related physical activities on the risk of incident and recurrent myocardial infarction and interaction with traffic-related air pollution: A cohort study. *J Am Heart Assoc* Published Online First: 2018. doi:10.1161/JAHA.118.009554

17 Zhang Z, Hoek G, Chang L yun, *et al.* Particulate matter air pollution, physical activity and systemic inflammation in Taiwanese adults. *Int J Hyg Environ Health* Published Online First: 2018. doi:10.1016/j.ijheh.2017.10.001

18 Norberg M, Wall S, Boman K, *et al.* The Västerbotten Intervention Programme: background, design and implications. *Glob Health Action* Published Online First: 2010. doi:10.3402/gha.v3i0.4643

19 Segersson D, Eneroth K, Gidhagen L, *et al.* **Health impact of PM10, PM2.5 and black carbon exposure due to different source sectors in Stockholm, Gothenburg and Umea,** Sweden. *Int J Environ Res Public Health* Published Online First: 2017. doi:10.3390/ijerph14070742

20 Hausberger S, Rexeis M, Zallinger M, *et al.* Emission Factors from the Model PHEM for the HBEFA Version 3. 2009.

21 Omstedt G, Bringfelt B, Johansson C. A model for vehicle-induced non-tailpipe emissions of particles along Swedish roads. *Atmos Environ* Published Online First: 2005. doi:10.1016/j.atmosenv.2005.06.037

22 SMHI. Airviro technical specification, appendix E. 2010. http://www.smhi.se/airviro/2.867/specification/

23 Andersen ZJ, de Nazelle A, Mendez MA, *et al.* A study of the combined effects of physical activity and air pollution on mortality in elderly urban residents: The Danish Diet, Cancer, and Health Cohort. *Environ Health Perspect* 2015.

24 Abramson N, Melton B. Leukocytosis: Basics of clinical assessment. Am. Fam. Physician. 2000.

25 World Health Organization. Review of evidence on health aspects of air pollution – REVIHAAP Project: Technical Report. 2013. doi:10.1007/BF00379640

For peer review only

A: Sensitivity analysis by excluding the individuals with follow-up time below the 25th percentile

Table 1. Hazard ratios (95% CI) for IHD associated with different exercise and commuting habits among persons with different air pollution exposure at home addresses.

Exercise in training clothes	Overall model with no interaction effects	Adjusted[a] HRs in categories of high and low particle exposure		Adjusted[a] interaction hazard ratio
		Low PM_{10}[b]	High PM_{10}[b]	Benefits of exercise/commuting comparing high and low particle exposure
Never	1	1	1	
≤ once/week	1.00 (0.84-1.19)	1.01 (0.84-1.21)	1.02 (0.85-1.23)	1.02 (0.79-1.31)
≥ twice/week	0.88 (0.76-1.02)	1.0 (0.77-1.31)	0.48 (0.34-0.7)	0.48 (0.31-0.76)
		Low PM2.5[c]	High PM2.5[c]	
Never		1	1	
≤ once/week		1.01 (0.84-1.21)	1.02 (0.85-1.23)	1.01 (0.78-1.31)
≥ twice/week		0.95 (0.72-1.24)	0.53 (0.37-0.75)	0.56 (0.36-0.87)
Active commuting per season		Low PM10[b]	High PM10[b]	
Non-active commuting	1	1	1	
≤ two seasons of four	1.02 (0.89-1.16)	1.16 (0.92-1.46)	0.85 (0.66-1.09)	0.73 (0.52-1.03)
> two seasons of four	0.74 (0.6-0.92)	0.91 (0.74-1.12)	0.83 (0.68-1.02)	0.91 (0.69-1.2)
		Low PM2.5[c]	High PM2.5[c]	
Non-active commuting		1	1	
≤ two seasons of four		1.18 (0.93-1.48)	0.84 (0.65-1.08)	0.70 (0.50-0.98)
> two seasons of four		0.98 (0.80-1.2)	0.79 (0.64-0.96)	0.82 (0.62-1.09)

[a]Adjusted for sex, calendar year, education, smoking, alcohol intake, occupation, neighbourhood mean income, leisure time physical activity, active commuting
[b]Low PM_{10}: ≤9.6 μg/m^3; High PM_{10}: >9.6 μg/m^3
[c]Low $PM_{2.5}$ ≤5.7 μg/m^3; High $PM_{2.5}$: >5.7 μg/m^3

Table 2. Hazard ratios (95% CI) for IHD associated with high air pollution levels (vs low) at home address among persons with different exercise/commuting habits.

	Overall model with No interaction effects	Adjusted[a] HRs in different exercise categories		
		Exercise in training clothes		
		Never	≤ once/week	≥ twice/week
Low PM_{10}[b]	1	1	1	1
High PM_{10}[b]	1.22 (0.96-1.56)	1.25 (1.0-1.56)	1.27 (1.03-1.58)	0.61 (0.40-0.93)
Low $PM_{2.5}$[c]	1	1	1	1
High $PM_{2.5}$[c]	0.95 (0.74-1.21)	1.15 (0.92-1.43)	1.16 (0.94-1.44)	0.64 (0.42-0.97)
		Active commuting		
		Non-active commuting	≤ two seasons of four	> two seasons of four
Low PM_{10}[b]		1	1	1
High PM_{10}[b]		1.28 (1.04-1.57)	0.94 (0.68-1.29)	1.16 (0.9-1.5)
Low $PM_{2.5}$[c]		1	1	1
High $PM_{2.5}$[c]		1.23 (1.0-1.51)	0.88 (0.64-1.21)	0.99 (0.76-1.27)

[a]Adjusted for sex, calendar year, education, smoking, alcohol intake, occupation, neighbourhood mean income, leisure time physical activity, active commuting
[b]Low PM_{10}: ≤9.6 μg/m^3; High PM_{10}: >9.6 μg/m^3
[c]Low $PM_{2.5}$ ≤5.7 μg/m^3; High $PM_{2.5}$: >5.7 μg/m^3

For peer review only

B1: Distributions of PM10 and PM2.5 moving averages (lag 1-5) for the person-years included in the study

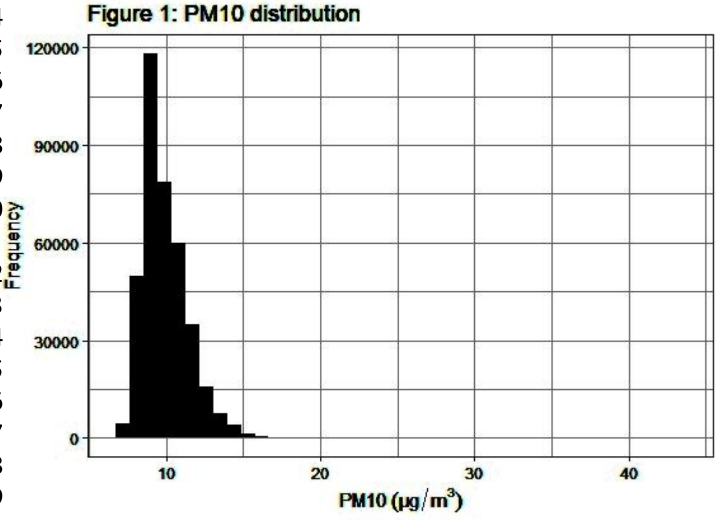

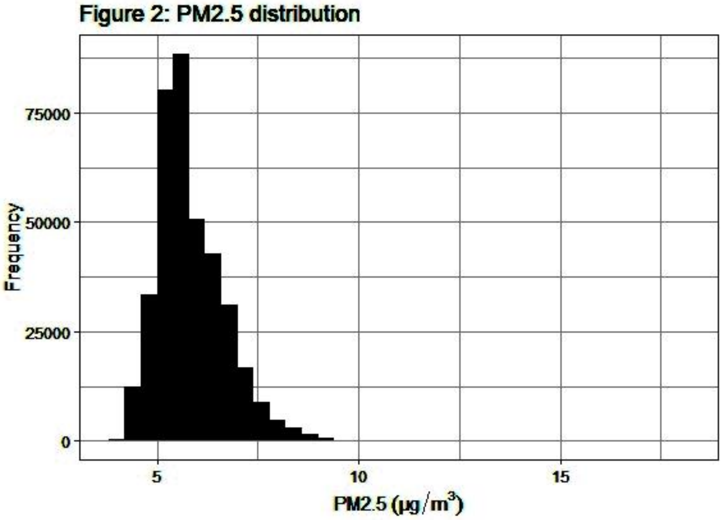

B2: Distributions of PM10 and PM2.5 moving averages (lag 1-5) for the person-years included in the study at different levels of physical activity (exercise in training clothes).

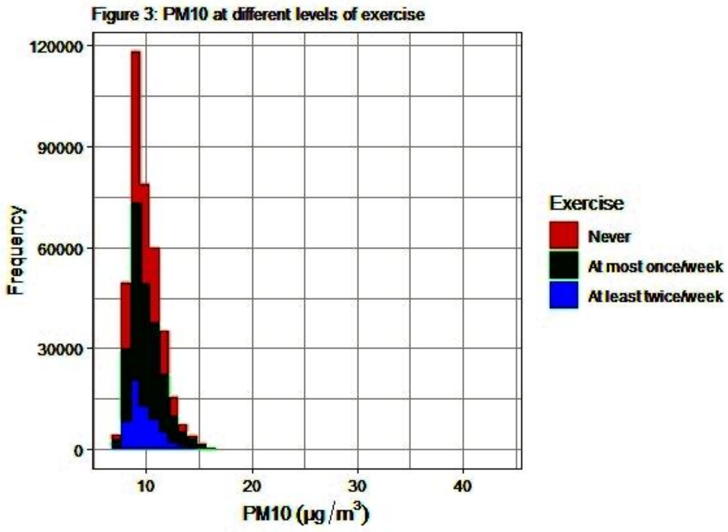

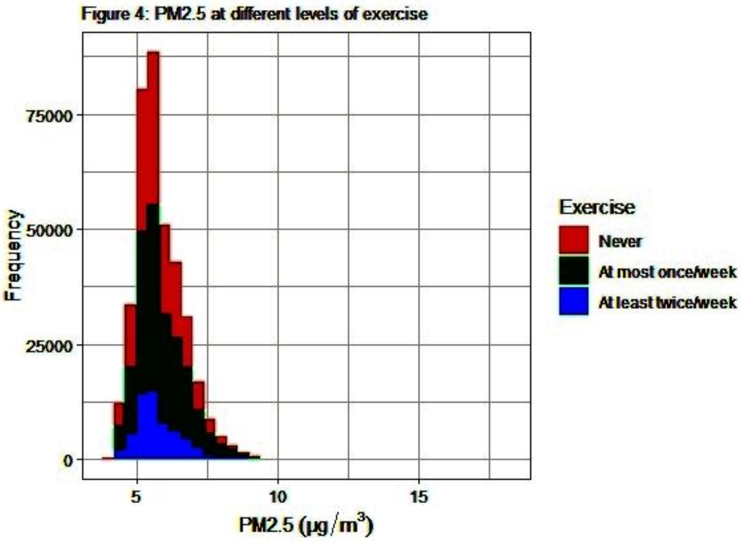

For peer review only

STROBE 2007 (v4) Statement—Checklist of items that should be included in reports of *cohort studies*

Section/Topic	Item #	Recommendation	Reported on page #
**Title and abstract**	1	(*a*) Indicate the study's design with a commonly used term in the title or the abstract	1
		(*b*) Provide in the abstract an informative and balanced summary of what was done and what was found	2
**Introduction**			
Background/rationale	2	Explain the scientific background and rationale for the investigation being reported	4
Objectives	3	State specific objectives, including any prespecified hypotheses	4
**Methods**			
Study design	4	Present key elements of study design early in the paper	5
Setting	5	Describe the setting, locations, and relevant dates, including periods of recruitment, exposure, follow-up, and data collection	5
Participants	6	(*a*) Give the eligibility criteria, and the sources and methods of selection of participants. Describe methods of follow-up	5
		(*b*) For matched studies, give matching criteria and number of exposed and unexposed	
Variables	7	Clearly define all outcomes, exposures, predictors, potential confounders, and effect modifiers. Give diagnostic criteria, if applicable	5-6
Data sources/ measurement	8*	For each variable of interest, give sources of data and details of methods of assessment (measurement). Describe comparability of assessment methods if there is more than one group	5-6
Bias	9	Describe any efforts to address potential sources of bias	6
Study size	10	Explain how the study size was arrived at	5
Quantitative variables	11	Explain how quantitative variables were handled in the analyses. If applicable, describe which groupings were chosen and why	5-6
Statistical methods	12	(*a*) Describe all statistical methods, including those used to control for confounding	7
		(*b*) Describe any methods used to examine subgroups and interactions	7
		(*c*) Explain how missing data were addressed	5
		(*d*) If applicable, explain how loss to follow-up was addressed	7
		(*e*) Describe any sensitivity analyses	
**Results**			

For peer review only

Participants	13*	(a) Report numbers of individuals at each stage of study—eg numbers potentially eligible, examined for eligibility, confirmed eligible, included in the study, completing follow-up, and analysed	5
		(b) Give reasons for non-participation at each stage	5
		(c) Consider use of a flow diagram	
Descriptive data	14*	(a) Give characteristics of study participants (eg demographic, clinical, social) and information on exposures and potential confounders	7
		(b) Indicate number of participants with missing data for each variable of interest	8
		(c) Summarise follow-up time (eg, average and total amount)	7
Outcome data	15*	Report numbers of outcome events or summary measures over time	7
Main results	16	(*a*) Give unadjusted estimates and, if applicable, confounder-adjusted estimates and their precision (eg, 95% confidence interval). Make clear which confounders were adjusted for and why they were included	8-11
		(*b*) Report category boundaries when continuous variables were categorized	9, 11
		(*c*) If relevant, consider translating estimates of relative risk into absolute risk for a meaningful time period	
Other analyses	17	Report other analyses done—eg analyses of subgroups and interactions, and sensitivity analyses	9-11
**Discussion**			
Key results	18	Summarise key results with reference to study objectives	12
**Limitations**			
Interpretation	20	Give a cautious overall interpretation of results considering objectives, limitations, multiplicity of analyses, results from similar studies, and other relevant evidence	12-13
Generalisability	21	Discuss the generalisability (external validity) of the study results	13
**Other information**			
Funding	22	Give the source of funding and the role of the funders for the present study and, if applicable, for the original study on which the present article is based	14

*Give information separately for cases and controls in case-control studies and, if applicable, for exposed and unexposed groups in cohort and cross-sectional studies.

Note: An Explanation and Elaboration article discusses each checklist item and gives methodological background and published examples of transparent reporting. The STROBE checklist is best used in conjunction with this article (freely available on the Web sites of PLoS Medicine at http://www.plosmedicine.org/, Annals of Internal Medicine at http://www.annals.org/, and Epidemiology at http://www.epidem.com/). Information on the STROBE Initiative is available at www.strobe-statement.org.

For peer review only

BMJ Open

Air pollution, physical activity and ischemic heart disease - A prospective cohort study of interaction effects

Journal:	*BMJ Open*
Manuscript ID	bmjopen-2020-040912.R2
Article Type:	Original research
Date Submitted by the Author:	21-Mar-2021
Complete List of Authors:	Raza, Wasif; Umeå Universitet Medicinska fakulteten, Public Health and Clinical Medicine, Sustainable Health<br>Krachler, Benno; Umeå Universitet Medicinska fakulteten, Public Health and Clinical Medicine, Sustainable Health<br>Forsberg, Bertil; Umeå Universitet Medicinska fakulteten, Public Health and Clinical Medicine, Sustainable health<br>Sommar, Johan; Umeå Universitet Medicinska fakulteten, Public Health and Clinical Medicine, Sustainable health
**Primary Subject Heading**:	Public health
Secondary Subject Heading:	Epidemiology
Keywords:	Ischaemic heart disease < CARDIOLOGY, PUBLIC HEALTH, Coronary heart disease < CARDIOLOGY

SCHOLARONE™
Manuscripts

I, the Submitting Author has the right to grant and does grant on behalf of all authors of the Work (as defined in the below author licence), an exclusive licence and/or a non-exclusive licence for contributions from authors who are: i) UK Crown employees; ii) where BMJ has agreed a CC-BY licence shall apply, and/or iii) in accordance with the terms applicable for US Federal Government officers or employees acting as part of their official duties; on a worldwide, perpetual, irrevocable, royalty-free basis to BMJ Publishing Group Ltd ("BMJ") its licensees and where the relevant Journal is co-owned by BMJ to the co-owners of the Journal, to publish the Work in this journal and any other BMJ products and to exploit all rights, as set out in our licence.

The Submitting Author accepts and understands that any supply made under these terms is made by BMJ to the Submitting Author unless you are acting as an employee on behalf of your employer or a postgraduate student of an affiliated institution which is paying any applicable article publishing charge ("APC") for Open Access articles. Where the Submitting Author wishes to make the Work available on an Open Access basis (and intends to pay the relevant APC), the terms of reuse of such Open Access shall be governed by a Creative Commons licence – details of these licences and which Creative Commons licence will apply to this Work are set out in our licence referred to above.

Other than as permitted in any relevant BMJ Author's Self Archiving Policies, I confirm this Work has not been accepted for publication elsewhere, is not being considered for publication elsewhere and does not duplicate material already published. I confirm all authors consent to publication of this Work and authorise the granting of this licence.

For peer review only

Air pollution, physical activity and ischemic heart disease - A prospective cohort study of interaction effects

Wasif Raza [1,*], Benno Krachler [1], Bertil Forsberg [1] and Johan Nilsson Sommar [1]

[1] Department of Public Health and Clinical Medicine, Section of Sustainable Health, Umeå University, Sweden; wasif.raza@umu.se (W.R.); benno.krachler@umu.se (B.K.); bertil.forsberg@umu.se (B.F.); johan.sommar@umu.se (J.N.S.)

* Correspondence: wasif.raza@umu.se ; Tel.: +46-72-289-5144

For peer review only

Abstract

Objective: To assess a possible interaction effect between physical activity and air pollution on first incidence of ischemic heart disease (IHD).

Design: Prospective cohort study

Setting: Umeå, Northern Sweden

Participants: We studied 34,748 adult participants of Västerbotten Intervention Programme cohort from 1990 to January 2014. Annual particulate matter concentrations ($PM_{2.5}$ and PM_{10}) at the participants' residential addresses were modelled and a questionnaire on frequency of exercise and active commuting was completed at baseline. Cox proportional hazards modelling was used to estimate 1) association with physical activity at different levels of air pollution, and 2) the association with particulate matter at different levels of physical activity.

Outcome: First incidence of IHD.

Results: Over a mean follow-up of 12.4 years, there were 1,148 IHD cases. Overall, we observed an increased risk of IHD among individuals with higher concentrations of particles at their home address. Exercise at least twice a week was associated with a lower risk of IHD among participants with high residential $PM_{2.5}$ (0.60; 95% CI: 0.44-0.82) and PM_{10} (0.55; 95% CI: 0.4-0.76). The same beneficial effect was not observed with low residential $PM_{2.5}$ (0.94; 95% CI: 0.72-1.22) and PM_{10} (0.99; 95% CI: 0.76-1.29). An increased risk associated with higher long-term exposure to particles was only observed among participants never exercising in training clothes and among those not performing any active commuting. However, only the interaction effect on hazard ratios for exercise was statistically significant.

Conclusion: Exercise was associated with a lower risk of first incidence of IHD among individuals with higher residential particle concentrations. An air pollution-associated risk was only observed among those who exercised less. The findings support the promotion of physical activity and a mitigation of air pollution.

Key Words: Air pollution, $PM_{2.5}$, PM_{10}, exercise, interaction, ischemic heart disease

For peer review only

Strengths and limitations of this study

- This study simultaneously evaluated the impact of physical activity and air pollution and their interaction on first incident IHD cases in a population with relatively low level of air pollution concentrations.
- For air pollution exposure, this study used individual time varying exposures of annual mean concentrations during follow-up based on population address registries.
- Another strength is the prospective design and the availability of baseline data on several important confounders.
- No exposure-response assessment could be performed since the statistical power only allowed for the formation of two exposure categories.
- Differences in air pollution exposure during active commuting might cause biased estimates due to exposure misclassification.

For peer review only

1 Introduction

Cardiovascular disease (CVD) is the most important cause of morbidity and premature mortality worldwide, accounting for 422.3 million cases and 17.92 million deaths in 2015 [1]. There is solid evidence that inflammation is a key upstream pathogenic mechanism [2]. Ambient air pollution, generally comprising ozone, sulphur dioxide, nitrogen dioxide and particulate matter (PM), is a leading contributor to the global burden of disease and an important risk factor for morbidity and mortality. Particulate matter is often measured as $PM_{2.5}$, to represent particles with a diameter of 2.5 micrometres or less, and PM_{10}, to represent particles with a diameter of 10 micrometres or less. Ambient $PM_{2.5}$ exposure alone has been estimated to account for 4.2 million deaths in 2015, of which 1.5 million deaths were caused by ischemic heart diseases (IHD) [3]. The underlying mechanisms mediating the pathogenic impact of air pollution involve systemic oxidative stress and inflammation [4,5]. Physical activity is a salutogenic factor in numerous non-communicable chronic diseases (NCDs), with physical inactivity being responsible for 6% of the burden of disease from coronary heart disease [6,7]. The beneficial effects of physical activity include protection against low-grade inflammation by releasing anti-inflammatory substances, such as interleukin 6, from contracting muscles [8,9].

Rapid urbanization and increased use of motorized transport contribute to modern day problems such as traffic congestion, traffic related air pollution and lack of physical activity. Promotion of active transportation by changing mode of transport from car to cycling and walking are among the different strategies used to tackle these challenges [10–12]. As inflammation is a causative mechanism for cardiovascular disease, it is conceivable that the anti-inflammatory effects of physical activity may mitigate the harmful effects associated with exposure to air pollution. However, one major concern with physical activity in a polluted environment is the increased inhalation of particles due to an increase in respiratory volume that may counteract the beneficial effects of physical activity [13,14].

The long-term effects of air pollution among individuals with different levels of leisure time physical activity have been estimated within the Danish Diet, Cancer, and Health cohort. The incidence of diabetes was assessed in relation to leisure time physical activity and nitrogen dioxide (NO_2) concentration at the home address. Residential NO_2 was found to be associated with increased incidence of diabetes, but only among physically active individuals [15]. As far as we know, only one study has examined the modifying effect of air pollution on the association between physical activity and cardiovascular disease. In their recent study, Kubesch and colleagues conclude that physical activity reduced the risk of first incidence of myocardial infarction (MI) and recurrent MI among individuals with high NO_2 concentration at the residential addresses [16]. As only one pollutant (NO_2) and only one cardiovascular outcome (MI) have been studied, the knowledge of a possible interaction between air pollution and physical activity on CVD is inconclusive.
A Taiwanese study found an independent inverse association of habitual physical activity with inflammation across different levels of $PM_{2.5}$ exposure, although long term exposure was associated with increased inflammation at all levels of physical activity [17].
We therefore aimed to examine interaction effects between physical activity and long-term exposure to $PM_{2.5}$ and PM_{10} at residential addresses on the incidence of ischemic heart disease (IHD). We wanted to assess: 1) whether air pollution modifies the beneficial effects of physical activity on IHD, and 2) whether physical activity modifies the harmful effects of air pollution on IHD.

2 Methods

To determine the interaction effect between air pollution and physical activity on IHD incidence, we combined cohort data which comprised risk factors for IHD, national registry data on IHD incidence from the Swedish National Board of Health and Welfare, and yearly annual mean air pollution particle concentrations at the individuals' residential addresses using dispersion models from the Swedish Clean Air and Climate Research Program (SCAC).

VIP (Västerbotten Intervention Programme) is a population based screening and prospective cohort study, developed to reduce the risk of future CVD and diabetes by promoting a healthy lifestyle among individuals living in the Västerbotten region. The SCAC developed methods to estimate exposure to source-specific particulate matter such as $PM_{2.5}$ and PM_{10} and at residential addresses in Gothenburg, Stockholm and Umeå.

2.1 Study population

VIP is an ongoing population-based health investigation survey of all individuals at ages 40, 50 or 60 years, depending upon risk factors, living in the Västerbotten region, who are invited to participate in systematic risk factor screening and individual counselling about healthy lifestyle habits. A detailed description of VIP has been presented elsewhere [18]. Between 1990 and 2014, a total of 42,488 of the VIP participants that lived in Umeå Municipality during the study period were included in the analysis. After exclusion of 7,740 participants with missing information on exercise, the study sample thus consisted of 34,748 individuals, 53% men and 47% women, 40-60 years of age at baseline examination, with no previous history of IHD at time of enrolment. After exclusion of individuals with missing information on included confounders the final number of included individuals were 31424 and 29218 for the analyses of exercise in training clothes and active commuting, respectively. All participants in the VIP gave their informed written consent, and the study was approved by the Regional Ethics Review Board at Umea University (DNR: 2014-136-32M and 2015/16-31Ö).

2.2 Leisure time exercise and active commuting

The VIP questionnaire includes various self-reported information on physical activity including frequency of leisure time physical activity and active commuting (cycling or walking to and from work). The association with IHD was assessed in relation to frequency of exercise in training clothes and amounts of active commuting. Exercise during the previous three months was categorized as never, rarely, once per week, 2-3 times per week or more than 3 times per week. Based on this information, participants were categorized as "Twice per week or more" if they exercised with a frequency of 2-3 times per week and more, "At most once a week" if they exercised rarely or once per week, and "Never" if no activity was performed. For active commuting, participants were asked about their mode of transport to work each season. Participants were classified in three categories: "Non- active commuting" if commuting every season by car or bus, "At most two seasons out of four" if cycling or walking at most half a year and "More than two seasons out of four", if cycling and walking more than half a year.

For peer review only - http://bmjopen.bmj.com/site/about/guidelines.xhtml

2.3 Covariates

The VIP questionnaire also gathered information on participants' educational status, occupation, smoking, alcohol intake and economic status. Education was defined according to the International Standard Classification of Education (ISCED), United Nations Educational, Scientific and Cultural Organization (UNESCO) 1997. Participants were asked about the highest level education they had achieved with eight predefined categories ranging from "pre-school" to "university education." Alcohol intake was assessed by the reported frequency of consumption with answering options that ranged from "Never" to "2-4 times/week." Information on smoking was gathered by using the question, "How often do you smoke?" Information on occupational status was obtained with a question, "What kind of job do you have nowadays?" with the answering options of eight predefined categories. Finally, information on occupation status was asked with the question, "What is your current occupation?" with 8 categories ranging from permanently employed to retired.

2.4 Air pollution concentrations

Annual mean total concentrations of $PM_{2.5}$ and PM_{10} for the years 1990-2011 (and thereafter linearly extrapolated up until year 2014) were obtained from the SCAC research program, described in detail elsewhere [19]. Briefly, concentrations of $PM_{2.5}$ and PM_{10} were estimated within SCAC by applying dispersion models on local or regional emission inventories. These emission inventories contain detailed information on emissions from different source categories, such as road traffic exhaust, road traffic non-exhaust, domestic heating, shipping and industrial activities. For Umeå, inventories were validated through monitoring for consistency. Emissions from small-scale residential heating were assessed using registry data from chimney sweepers that included the type of wood stove or boiler. The addresses of these residences were geocoded using the geographical centre coordinate of the estate. Road traffic emission factors for PM exhaust for different vehicle types, speeds and driving conditions were calculated based on the Handbook Emission Factors for Road Transport (HBEFA) version 3.1 [20]. Estimates for non-exhaust contribution from brake and tire wear were based on Omstedt et al. [21]. The annual average emission from shipping was used in the modelling on a 1x1 km^2 grid resolution. The emissions from other sources such as industrial processes, off-road machinery and agriculture was collected from Swedish Meteorological and Hydrological Institute (SMHI). To obtain annual average emissions of $PM_{2.5}$ and PM_{10}, Gaussian models included in the Airviro air quality management system (SMHI, 2010) were used for simulation based on hourly meteorological data for 1990, 2000 and 2011 [22]. The comparison between measured and modelled $PM_{2.5}$ and PM_{10} agreed well at most monitoring stations (r^2=0.87 and r^2=0.65, respectively).

2.5 Outcome

We linked the records in VIP and through the unique Swedish personal identification number with data on first IHD event cases from the National Patient Register and the Cause of Death Register, both at the National Board of Health and Welfare, using primary discharge diagnoses for IHD according to International Classification of Diseases, 10th revision (ICD-10): code I20-125.

For peer review only

2.6 Patient and public involvement

No patients were involved in this study.

2.7 Statistical Analysis

We performed survival analyses using Cox regression proportion hazard model to estimate hazard ratios (HRs), and 95% confidence intervals (CIs), to estimate 1) the association between first incident IHD and air pollution exposure at different levels of physical activity and 2) the association between first incident IHD and physical activity at different levels of air pollution exposure. Age was used as the underlying timescale since it is a stronger confounder than calendar time. Follow-up started at date of recruitment to the cohort and ended with the earliest of the date of first IHD case, emigration, death, or 31 December 2013. Interaction between physical activity and air pollution and their impact on IHD was studied by introducing an interaction term into the model. Residential annual mean particle concentrations were used to calculate moving averages over the recent five years which were thereafter categorized as below or above the median concentration for $PM_{2.5}$ and PM10, respectively. Sensitivity analyses were also performed with PM concentrations categorized by tertiles. Interaction with physical activity was assessed based on 1) the frequency of exercise in training clothes and 2) the number of seasons the individual walked or cycled to work. Active commuting by walking or cycling was categorized into three groups: non-active commuters, active commuters at most half of the year (up to two out of four seasons), active commuters more than half a year (more than two out of four seasons). Estimates were adjusted for a pre-specified set of covariates: calendar year as a penalized cubic spline with 3 degrees of freedom, gender (male vs female), highest education level (compulsory, high school, university), alcohol intake (never, once/month or sometimes, 2-4 times/month, 2-3 times/week, ≥4 times/week), smoking (previous non-regular smoker, non-regular smoker, cigarette smoker, cigar or pipe smoker), occupation (gainfully employed, unemployed, not gainfully employed, retired), and registry data on area level mean income year 1994. In the basic model we adjusted only for gender and exposure year. All analyses were performed using R version 3.4.2, and the statistical inference was conducted with a 5% significance level. T-tests, global analysis of variance tests (ANOVA) and chi-square tests were used to test for differences in means and proportions of covariates between categories of exercise in training clothes (Table 1). The Schoenfeld residuals test was used to assess the assumption of proportional hazards. A sensitivity analysis was conducted by excluding participants with follow-up time below the 25th percentile.

3 Results

The mean age at recruitment to this cohort was 45.8 years. Among the 34,748 participants 1,148 cases of IHD were identified during a mean follow-up time of 12.4 years. Of those cases, 500 never exercised, 529 exercised at most once a week and 119 exercised at least twice a week. Table 1 summarizes characteristics of participants according to different levels of leisure time physical activity. Participants not reporting any leisure time exercise were older and more likely to be male, non-commuters and to belong to a lower socioeconomic group. Subjects performing moderate to high-level physical activity,

For peer review only - http://bmjopen.bmj.com/site/about/guidelines.xhtml

were more likely to be women, non-smokers, and active commuters. The 5-year means of PM_{10} and $PM_{2.5}$ concentrations were different between leisure time physical activity categories, with at most 7 and 6% difference respectively for PM_{10} and $PM_{2.5}$ (with distributions presented in Supplemental material A: Figures 1-4).

For peer review only

Table 1: Characteristics of participants at different levels of exercise in training clothes at baseline

Characteristics	Never	At most once a week	Twice per week or more	p
Exercise in training clothes n (%)	13043 (37.5)	14994 (43.2)	6711 (19.3)	
PM_{10}, µg/m^3 (mean (SD))	10.93 (2.00)	11.06 (1.96)	10.39 (1.94)	<0.001
$PM_{2.5,}$ µg/m^3 (mean (SD))	6.41 (1.12)	6.49 (1.12)	6.11 (1.11)	<0.001
Frequency of active commuting (%)				<0.001
Non-active commuting	6369 (48.8)	6409 (42.7)	2666 (39.7)	
At most two seasons of four	1806 (13.8)	2547 (17.0)	1037 (15.5)	
More than two seasons of four	3506 (26.9)	4976 (33.2)	2555 (38.1)	
Missing	1362 (10.4)	1062 (7.1)	453 (6.8)	
Alcohol intake (%)				<0.001
Never	135 (1.0)	120 (0.8)	79 (1.2)	
Once/month or sometimes	5337 (40.9)	5950 (39.7)	2790 (41.6)	
2-4 times/month	2000 (15.3)	2436 (16.2)	1061 (15.8)	
2-3 times/week	97 (0.7)	93 (0.6)	54 (0.8)	
≥ 4 times/week	5068 (38.9)	5861 (39.1)	2265 (33.8)	
Missing	406 (3.1)	534 (3.6)	462 (6.9)	
Smoking (%)				<0.001
Never smoker	5139 (39.4)	7240 (48.3)	3467 (51.7)	
Previous non-regular smoker	1072 (8.2)	1458 (9.7)	711 (10.6)	
Non-regular smoker	581 (4.5)	807 (5.4)	348 (5.2)	
Previous regular smoker	2729 (20.9)	2865 (19.1)	1201 (17.9)	
Cigarette smoker	2789 (21.4)	1805 (12.0)	426 (6.3)	
Cigar or pipe smoker	186 (1.4)	130 (0.9)	45 (0.7)	
Missing	547 (4.2)	689 (4.6)	513 (7.6)	
Highest education level (%)				<0.001
Compulsory	5534 (42.4)	4319 (28.8)	1369 (20.4)	
High	3573 (27.4)	4116 (27.5)	1951 (29.1)	
University	3449 (26.4)	5933 (39.6)	2911 (43.4)	
Missing	487 (3.7)	626 (4.2)	480 (7.2)	
Gender %, (men)	7072 (54.2)	8036 (53.6)	3274 (48.8)	<0.001
Age, y (mean (SD))	47.3 (9.1)	45.3 (9.0)	44.3 (8.6)	<0.001
Occupation (%)	<0.001			
Gainfully employed	10536 (80.8)	12690 (84.6)	5452 (81.2)	
Unemployed	478 (3.7)	382 (2.5)	174 (2.6)	
Not gainfully employed	318 (2.4)	352 (2.3)	160 (2.4)	
Retired	673 (5.2)	449 (3.0)	194 (2.9)	
Missing	1038 (8.0)	1121 (7.5)	731 (10.9)	
Mean income for the neighbourhood (SEK*) (mean (SD))	128286 (23018)	130332 (23875)	130222 (24606)	<0.001

*SEK=Swedish krona

Compared with individuals who reported no exercise, those participants that exercised at least twice per week had a 24% lower risk of IHD (Table 2). The corresponding overall estimate associated with active commuting was a 13% reduced risk of IHD among individuals commuting more than two seasons per year.

Allowing for an interaction between the frequency of exercise in training clothes and particle concentrations (PM_{10} and $PM_{2.5}$) at the home address, the average 24% risk reduction from exercising at least twice per week was found to be driven by statistically significant interaction between exercise and particle exposure with 45% and 40% risk reduction among individuals with high PM_{10} and $PM_{2.5}$ concentrations, respectively (table 2). The interaction coefficients estimating the additional benefit of exercise among individuals with a high PM_{10} concentration at their home addresses were a 3% increased risk among those who exercised at most once a week whereas a decreased risk of 44% was estimated among those who exercised at least twice a week. The corresponding estimates among those with high $PM_{2.5}$ concentrations were a risk reduction of 0% and 36% respectively.

For active commuters with low particle exposure at their home address, the risk of incident IHD was 17% and 18% higher among those commuting one or two seasons per year and 7% and 3% lower among those commuting at least two seasons per year, for PM_{10} and $PM_{2.5}$ respectively. The benefit of active commuting was larger among individuals with a high particle concentration at their home address: risk reductions for active commuting during one or two seasons were 12% and 13% and for more than two seasons 18% and 21% respectively, compared with non-active commuters. No statistically significant interaction was found between active commuting and particle concentrations at the home address.

For peer review only

Table 2: Hazard ratios (95% CI) for IHD associated with different exercise and commuting habits among persons with different air pollution exposure at home addresses.

Exercise in training clothes	Overall model with no interaction effects	Proportional hazard p[d]-value *	Adjusted[a] HRs in categories of high and low particle exposure				Adjusted[a] interaction hazard ratio	
			Low PM_{10}[b]	Proportional hazard p-value[d]	High PM_{10}[b]	Proportional hazard p-value[d]	Benefits of exercise/commuting comparing high and low particle exposure	Proportional hazard p-value[d]
Never	1		1		1			
≤ once/week	1.03 (0.90-1.16)	0.35	1.01 (0.84-1.21)	0.67	1.04 (0.88-1.23)	0.35	1.03 (0.81-1.32)	0.75
≥ twice/week	0.76 (0.62-0.93)	0.08	0.99 (0.76-1.29)	0.47	0.55 (0.40-0.76)	0.24	0.56 (0.37-0.84)	0.65
			Low $PM_{2.5}$[c]		High $PM_{2.5}$[c]			
Never			1		1			
≤ once/week			1.03 (0.86-1.23)	0.78	1.03 (0.87-1.22)	0.30	1.00 (0.78-1.28)	0.61
≥ twice/week			0.94 (0.72-1.22)	0.69	0.60 (0.44-0.82)	0.09	0.64 (0.43-0.96)	0.30
Active commuting per season			Low PM_{10}[b]		High PM_{10}[b]			
Non-active commuting	1		1		1			
≤ two seasons of four	1.01 (0.86-1.19)	0.50	1.17 (0.93-1.47)	0.35	0.88 (0.70-1.11)	0.70	0.76 (0.55-1.04)	0.34
> two seasons of four	0.87 (0.76-0.998)	0.85	0.93 (0.76-1.14)	0.48	0.82 (0.68-0.98)	0.89	0.88 (0.67-1.15)	0.53
			Low $PM_{2.5}$[c]		High $PM_{2.5}$[c]			
Non-active commuting			1		1			
≤ two seasons of four			1.18 (0.94-1.49)	0.43	0.87 (0.69-1.10)	0.76	0.73 (0.53-1.01)	0.43
> two seasons of four			0.97 (0.80-1.19)	0.37	0.79 (0.65-0.95)	0.89	0.81 (0.62-1.06)	0.44

[a]Adjusted for sex, calendar year, education, smoking, alcohol intake, occupation, neighbourhood mean income, leisure time physical activity, and active commuting

[b]Low PM_{10}: ≤9.6 µg/m^3; High PM_{10}: >9.6 µg/m^3

[c]Low $PM_{2.5}$ ≤5.7 µg/m^3; High $PM_{2.5}$: >5.7 µg/m^3

[d] p-value of the Schoenfeld residual test of proportional hazards

For peer review only

For peer review only - http://bmjopen.bmj.com/site/about/guidelines.xhtml

Individuals exposed to high concentrations of PM_{10} and $PM_{2.5}$ at their home address had a 14% and 1% increased risk of incident IHD respectively, compared to individuals with low concentrations (Table 3). These increased risks were, however, not statistically significant.

When including an interaction between particle concentrations and exercise, risk estimates showed a positive association between air pollution and IHD among individuals performing no exercise and those exercising no more than once a week, whereas a negative association was found among those exercising more than twice a week (Table 3).

Compared with individuals with low residential particle concentration, a high concentration of PM_{10} was associated with 21% and 25% increased risk for IHD for those who never exercised in training clothes and those who exercised at most once a week, respectively, and showed a 32% decreased risk for those exercising at least twice a week; only the association for those exercising at most once a week was statistically significant. The corresponding estimates associated with $PM_{2.5}$ are increased risk of IHD of 16% for those never exercising and those who exercised at most once a week, and a decreased risk of 26% among those exercising at least twice a week (Table 3); none of these associations was statistically significant.

IHD risk associated with high residential PM_{10} and $PM_{2.5}$ compared with low residential particle concentration was found to be 26% and 24% higher, respectively, among those never actively commuting; both these results were statistically significant. Among those actively commuting one or two seasons per year, IHD risk was 5% and 9% lower, respectively, while among those actively commuting more than two seasons out of four, the risk was 10% and 1% higher, respectively; none of these risks were, however, statistically significantly different from the air pollution-associated risks among the active commuters (Table 2). Overall, no statistically significant modifying effect of exercise and active commuting on the association between high particle concentration at home addresses and IHD was observed (Table 3).

Sensitivity analyses

Excluding participants with short follow-up time (below the 25th percentile of 3.4 years) did not affect the main conclusions of our study, however estimates tended to be lower for overall effect of air pollution on IHD (Supplementary material B: Tables 1 and 2).

Sensitivity analyses were also conducted with PM concentrations in tertiles. An indication of a dose-response was found, with increasing benefits of exercise in training clothes with higher levels of PM concentrations at the home address (Supplementary material C: Table 3). Exercising at least twice per week (compared with never) reduced the risk of incident IHD by 5, 17, and 49% within the first, second and third tertile of PM10 exposure, respectively. Similar risk reductions were found in relation to tertiles of PM2.5.

No such interaction dose-response was however found for risks associated with PM exposure. Risk estimates associated with PM were somewhat higher among individuals that exercised once a week compared those who never exercised, whereas no increased risk associated with either PM10 or PM2.5 was found among individuals that exercised at least twice per week (Supplementary material C: Table 4). A dose-response with increasing risks for IHD were found with both PM10 and PM2.5 among those that never exercised.

For peer review only

These interactions between PM concentrations and exercise at least twice per week (compared with never) were statistically significant for PM10 and borderline statistically significant for PM2.5 (Supplementary material C: Table 5).

For peer review only

Table 3: Hazard ratios (95% CI) for IHD associated with high air pollution levels (vs low) at home address among persons with different exercise/commuting habits.

	Overall model with no interaction effects	Proportional hazard p-value[d]	Adjusted[a] HRs in categories of high and low particle exposure					
			Exercise in training clothes					
			Never	Proportional hazard p-value[d]	≤ once/week	Proportional hazard p-value[d]	≥ twice/week	Proportional hazard p-value[d]
Low PM_{10}[b]	1		1		1		1	
High PM_{10}[b]	1.14 (0.9-1.45)	0.55	1.21 (0.97-1.49)	0.71	1.25 (1.01-1.54)	0.45	0.68 (0.46-0.998)	0.50
Low $PM_{2.5}$[c]	1		1		1		1	
High $PM_{2.5}$[c]	1.01 (0.8-1.28)	1.10	1.16 (0.94-1.44)	0.38	1.16 (0.95-1.43)	0.13	0.74 (0.51-1.09)	0.11
			Active commuting					
			Non-active commuting		≤ two seasons of four	P-value	> two seasons of four	
Low PM_{10}[b]			1		1		1	
High PM_{10}[b]			1.26 (1.03-1.54)	0.39	0.95 (0.7-1.29)	0.66	1.10 (0.86-1.41)	0.17
Low $PM_{2.5}$[c]			1		1		1	
High $PM_{2.5}$[c]			1.24 (1.02-1.51)	0.11	0.91 (0.67-1.24)	0.84	1.01 (0.79-1.29)	0.04

[a]Adjusted for sex, calendar year, education, smoking, alcohol intake, occupation, neighbourhood mean income, leisure time physical activity, active commuting

[b]Low PM_{10}: ≤9.6 μg/m^3; High PM_{10}: >9.6 μg/m^3

[c]Low $PM_{2.5}$ ≤5.7 μg/m^3; High $PM_{2.5}$: >5.7 μg/m^3

[d] p-value of the Schoenfeld residual test of proportional hazards

For peer review only

4 Discussion

Overall, we found increased risk of first incident IHD associated with air pollution at the home address but a protective effect of physical activity. A statistically significant beneficial effect of exercise was found among individuals with high PM10/PM2.5, but not among individuals with low levels. Also, for active commuting the benefits were greater among individuals with high residential particle concentrations, but these differences were not statistically significant. Air pollution concentration-associated risks were found among individuals who exercise at most once a week but not among individuals exercising at least twice a week. Statistically significantly increased risks were also found among non-active commuters. These risks were, however, not statistically significantly different from air pollution-associated risks among the active commuters.

Our findings are in accordance with a longitudinal cohort study on MI within the Danish Diet, Cancer, and Health cohort which found an increased benefit of participation in sports among individuals with high NO_2 concentration at the home addresses [16]. The reported risk reduction was 9, 15, and 24% respectively among individuals with low (<14.3 µg/m3), medium (14.3-21 µg/m3) and high (>21 µg/m3) residential NO_2 concentration. For walking and cycling they also estimated greater risk reductions for first incident MI among individuals with higher air pollution concentrations. In the same Danish cohort, the long term benefits of physical activity on CVD mortality were also found to be greater among individuals with high residential NO_2 [23]. The risk reduction associated with participation in cycling and gardening among individuals exposed to high residential NO_2 (≥ 19 µg/m3) was greater than those exposed to moderate/low NO_2 concentration (<19 µg/m3). Among participants exposed to high NO_2, the risk reduction for cycling and gardening was 30% and 23%, respectively, whereas among participants exposed to low NO_2, risk reduction was 17% and 15%, respectively. However, the interaction effects in these two studies were not statistically significant.

Opposite findings were observed for the modifying effect of physical activity on the association between air pollution and the incidence of diabetes [15]. Among the participants in the Danish cohort, the risk of developing diabetes increased by 10% per interquartile range of 4.9 mg/m^3 residential NO_2 among physically active individuals, but there was no difference among less physically active individuals. The authors considered that this may be due to an additive rather multiplicative interaction, thus resulting in an increased risk estimate only among physical active individuals with a low risk of developing diabetes.

As inflammation is a causative mechanism for cardiovascular disease, we hypothesized that the anti-inflammatory effects of physical activity may reduce air pollution-associated risks since inflammation is one among several different pathways for the harmful health effects of air pollution. The findings of greater benefits of physical activity among individuals with higher air pollution exposure for incident IHD risk in our study and incident MI and CVD mortality in the Danish cohort, support such a hypothesis. However, a study on physical activity and white blood cell counts conducted in a large cohort of Taiwanese adults suggested no effect modification by residential air pollution measured as $PM_{2.5}$ [17]. Both physical activity and residential air pollution were, however, found to be associated with an inflammatory response assessed by white blood cell counts. However, the association between physical activity and white blood cell count is variable because exercise also causes a transient increase in white blood cell count which usually normalises within 24 hours [24]. The results of our study cannot be directly compared with the above mentioned studies due to the difference in pollutants [15,16,23] and health outcomes [15,16,23]. This study contributes with air pollution effect estimates on IHD incidence in a population with a relatively low level of air pollution concentrations. Compared with the previous

cohort studies on interaction effects between air pollution and physical activity, the annual mean $PM_{2.5}$ concentration was 3-4 times lower. The annual mean in the Taiwanese cohort was 27 μg/m3 and a recent study within the same population as the DHC-cohort studies reported 18 μg/m3. Even at these lower levels of air pollution an increased risk associated with air pollution exposure was found, however not among those who exercised at least twice a week. Even though active commuting may result in higher air pollution exposure compared with for instance driving a car to work, the risk of an IHD event was still reduced since the benefit of the physical activity was greater than the IHD risk imposed by the air pollution exposure.

A major strength of our study is the air pollution particle concentration exposure data since particles are considered to be the causal component of air pollution [25]. The study used individual time varying exposures of annual mean concentrations during follow-up based on population address registries. The dispersion model used for modelling of particle concentrations has previously been validated [19]. Within the DHC studies NO_2 was used as a proxy for traffic generated air pollution and was assessed only at residential addresses at the year of recruitment or as an annual mean during follow-up. A limitation of our exposure data is that the statistical power only allowed for two exposure categories and therefore no exposure-response assessment was performed.

Another strength of our study is the prospective design, the long follow-up period, the large cohort size and the availability of baseline data on several important confounders. A limitation is the lack of information on the intensity and duration of physical activity and therefore only frequency of exercise could be considered. The study also lacked information on changes in physical activity and other life-style factors during follow-up as the information was only retrieved at baseline.

There is a risk of reverse causation if individuals at their baseline examination had a low physical activity level due to poorer health. Individuals could for instance have diseases that affect their risk to later in life have an IHD event (such as diabetes) prior to baseline examination. If this prior disease also affected the frequency the individual exercised in training clothes, or mode of commuting, then a reverse causation between physical activity and IHD risk may occur. Individuals with a prior IHD event at baseline were however excluded, and the sensitivity analyses that excluded individuals with follow-up time below the 25th percentile showed that this did not change the results. Furthermore, we lacked information on whether exercise is taking place outdoors or indoors. For active commuters we also lack air pollution exposure calculations during the commute. This would cause exposure misclassification among active commuters with a higher in-traffic air pollution exposure dose compared with non-active commuters, causing a possible bias to the null. This would also occur if individuals chose not to exercise outside during times with high air pollution exposure.

For peer review only

5 Conclusion

The study estimated that exercise reduced the risk of first incident IHD, but only among individuals with higher residential particle concentrations (above median). Similarly, the harmful air pollution effect on IHD was only found among those who exercised less. Our results reinforce the public health message that physical activity is beneficial for cardiovascular health and thus support the adoption of strategies to improve health through promotion of physical activity and mitigation of air pollution. Further studies are needed to build on the evidence of physical activity and air pollution interactions on the incidence cardiovascular disease. Air pollution exposures during commuting should also be considered in these studies.

Author's contributions: All the authors contributed to the study conception and design. W.R. and J.N.S. analysed the data. W.R wrote the first draft of the manuscript. B.F., B.K. and J.N.S. critically revised the manuscript for important intellectual content. All authors read and approved the final version of the manuscript.

Funding: The study was funded by FORTE (Forskingsrådet för hälsa, arbetsliv och välfärd) [grant number 2012-1296], a Swedish state agency that finances scientific research in health, welfare, and working life, and by grants from Folksam research foundation and the Swedish state under the agreement between the Swedish government and the county councils (ALF) awarded B F [grant number: VLL-645781].

Competing interests: None.

Participants consent: Obtained.

Ethics approval: The study was approved by the Regional Ethics Review Board at Umea University (DNR: 2014-136-32M and 2015/16-31Ö).
Data availability: No additional data is available

For peer review only

6 References

1 Lelieveld J, Klingmüller K, Pozzer A, *et al.* Cardiovascular disease burden from ambient air pollution in Europe reassessed using novel hazard ratio functions. *Eur Heart J* Published Online First: 2019. doi:10.1093/eurheartj/ehz135

2 Shrivastava AK, Singh HV, Raizada A, *et al.* C-reactive protein, inflammation and coronary heart disease. Egypt. Hear. J. 2015. doi:10.1016/j.ehj.2014.11.005

3 Cohen AJ, Brauer M, Burnett R, *et al.* Estimates and 25-year trends of the global burden of disease attributable to ambient air pollution: an analysis of data from the Global Burden of Diseases Study 2015. *Lancet* Published Online First: 2017. doi:10.1016/S0140-6736(17)30505-6

4 Münzel T, Gori T, Al-Kindi S, *et al.* Effects of gaseous and solid constituents of air pollution on endothelial function. Eur. Heart J. 2018. doi:10.1093/eurheartj/ehy481

5 Brook RD, Rajagopalan S, Pope CA, *et al.* Particulate matter air pollution and cardiovascular disease: An update to the scientific statement from the American Heart Association. *Circulation* Published Online First: 2010. doi:10.1161/CIR.0b013e3181dbece1

6 Stevens G. Global Health Risks: Mortality and burden of disease attributable to selected major risks. World Heal. Organ. 2009;**87**:646–646. doi:10.2471/BLT.09.070565

7 Lee I, Shiroma EJ, Lobelo F, *et al.* Impact of Physical Inactivity on the World's Major Non-Communicable Diseases. *Lancet* Published Online First: 2012. doi:10.1016/S0140-6736(12)61031-9

8 Nimmo MA, Leggate M, Viana JL, *et al.* The effect of physical activity on mediators of inflammation. Diabetes, Obes. Metab. 2013. doi:10.1111/dom.12156

9 Petersen AMW, Pedersen BK. The anti-inflammatory effect of exercise. *J Appl Physiol* Published Online First: 2005. doi:10.1152/japplphysiol.00164.2004

10 Rajé F, Tight M, Pope FD. Traffic pollution: A search for solutions for a city like Nairobi. *Cities* Published Online First: 2018. doi:10.1016/j.cities.2018.05.008

11 Rojas-Rueda D, De Nazelle A, Teixidó O, *et al.* Health impact assessment of increasing public transport and cycling use in Barcelona: a morbidity and burden of disease approach. *Prev Med (Baltim)* 2013;**57**:573–9.

12 Maizlish N, Woodcock J, Co S, *et al.* Health cobenefits and transportation-related reductions in greenhouse gas emissions in the San Francisco Bay area. *Am J Public Health* 2013;**103**:703–9.

13 Tainio M, de Nazelle AJ, Götschi T, *et al.* Can air pollution negate the health benefits of cycling and walking? *Prev Med (Baltim)* Published Online First: 2015. doi:10.1016/j.ypmed.2016.02.002

14 Giles L V., Koehle MS. The health effects of exercising in air pollution. Sport. Med. 2014. doi:10.1007/s40279-013-0108-z

15 Andersen ZJ, Raaschou-Nielsen O, Ketzel M, *et al.* Diabetes incidence and long-term exposure to air pollution: A cohort study. *Diabetes Care* Published Online First: 2012. doi:10.2337/dc11-1155

For peer review only

16 Kubesch NJ, Jørgensen JT, Hoffmann B, *et al.* Effects of leisure-time and transport-related physical activities on the risk of incident and recurrent myocardial infarction and interaction with traffic-related air pollution: A cohort study. *J Am Heart Assoc* Published Online First: 2018. doi:10.1161/JAHA.118.009554

17 Zhang Z, Hoek G, Chang L yun, *et al.* Particulate matter air pollution, physical activity and systemic inflammation in Taiwanese adults. *Int J Hyg Environ Health* Published Online First: 2018. doi:10.1016/j.ijheh.2017.10.001

18 Norberg M, Wall S, Boman K, *et al.* The Västerbotten Intervention Programme: background, design and implications. *Glob Health Action* Published Online First: 2010. doi:10.3402/gha.v3i0.4643

19 Segersson D, Eneroth K, Gidhagen L, *et al.* **Health impact of PM10, PM2.5 and black carbon exposure due to different source sectors in Stockholm, Gothenburg and Umea,** Sweden. *Int J Environ Res Public Health* Published Online First: 2017. doi:10.3390/ijerph14070742

20 Hausberger S, Rexeis M, Zallinger M, *et al.* Emission Factors from the Model PHEM for the HBEFA Version 3. 2009.

21 Omstedt G, Bringfelt B, Johansson C. A model for vehicle-induced non-tailpipe emissions of particles along Swedish roads. *Atmos Environ* Published Online First: 2005. doi:10.1016/j.atmosenv.2005.06.037

22 SMHI. Airviro technical specification, appendix E. 2010. http://www.smhi.se/airviro/2.867/specification/

23 Andersen ZJ, de Nazelle A, Mendez MA, *et al.* A study of the combined effects of physical activity and air pollution on mortality in elderly urban residents: The Danish Diet, Cancer, and Health Cohort. *Environ Health Perspect* 2015.

24 Abramson N, Melton B. Leukocytosis: Basics of clinical assessment. Am. Fam. Physician. 2000.

25 World Health Organization. Review of evidence on health aspects of air pollution – REVIHAAP Project: Technical Report. 2013. doi:10.1007/BF00379640

For peer review only

A1: Distributions of PM10 and PM2.5 moving averages (lag 1-5) for the person-years included in the study

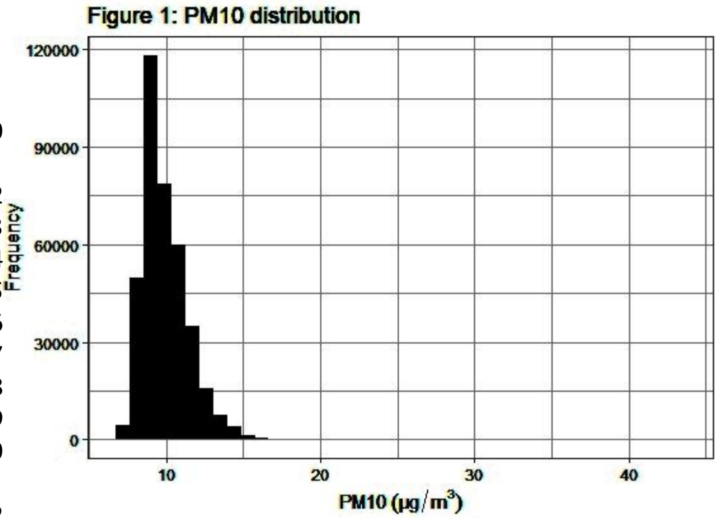

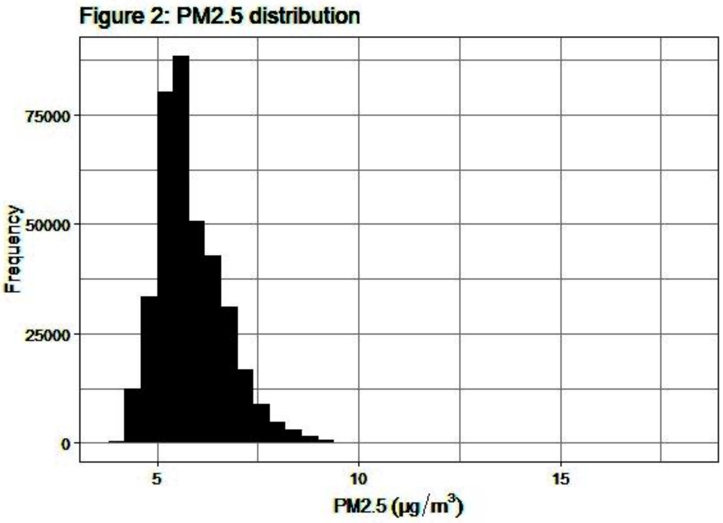

A2: Distributions of PM10 and PM2.5 moving averages (lag 1-5) for the person-years included in the study at different levels of physical activity (exercise in training clothes).

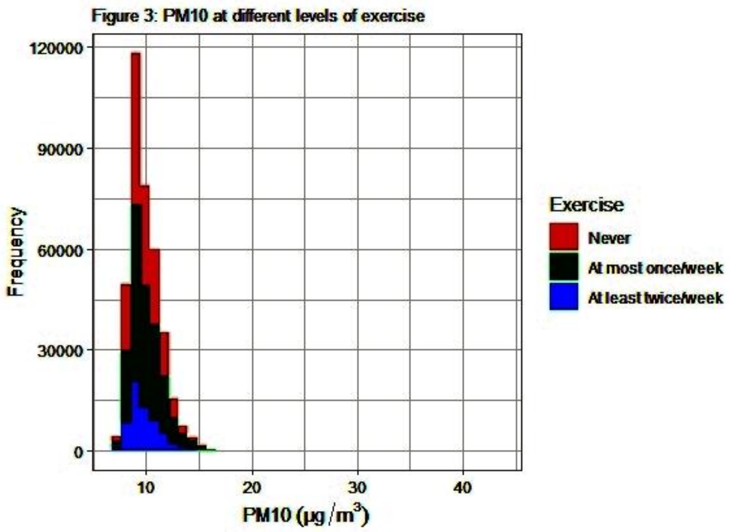

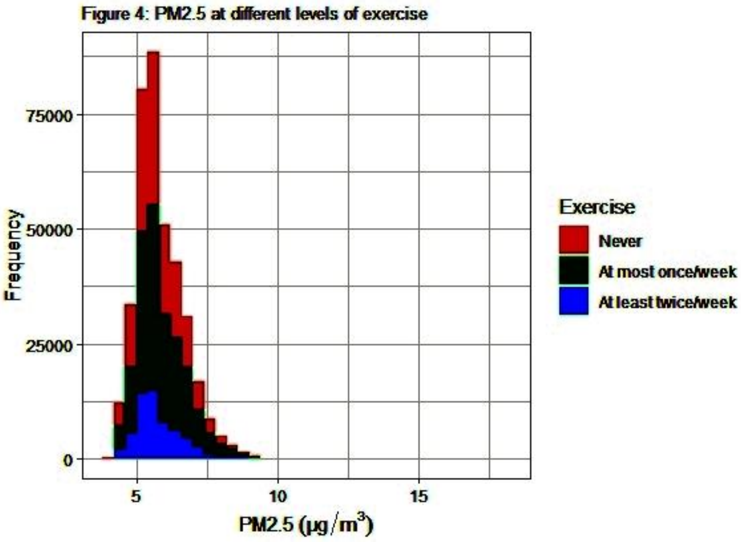

B: Sensitivity analysis by excluding the individuals with follow-up time below the 25th percentile

Table 1. Hazard ratios (95% CI) for IHD associated with different exercise and commuting habits among persons with different air pollution exposure at home addresses.

Exercise in training clothes	Overall model with no interaction effects	Adjusted[a] HRs in categories of high and low particle exposure		Adjusted[a] interaction hazard ratio
		Low PM_{10}[b]	High PM_{10}[b]	Benefits of exercise/commuting comparing high and low particle exposure
Never	1	1	1	
≤ once/week	1.00 (0.84-1.19)	1.01 (0.84-1.21)	1.02 (0.85-1.23)	1.02 (0.79-1.31)
≥ twice/week	0.88 (0.76-1.02)	1.0 (0.77-1.31)	0.48 (0.34-0.7)	0.48 (0.31-0.76)
		Low PM2.5[c]	High PM2.5[c]	
Never		1	1	
≤ once/week		1.01 (0.84-1.21)	1.02 (0.85-1.23)	1.01 (0.78-1.31)
≥ twice/week		0.95 (0.72-1.24)	0.53 (0.37-0.75)	0.56 (0.36-0.87)
Active commuting per season		Low PM10[b]	High PM10[b]	
Non-active commuting	1	1	1	
≤ two seasons of four	1.02 (0.89-1.16)	1.16 (0.92-1.46)	0.85 (0.66-1.09)	0.73 (0.52-1.03)
> two seasons of four	0.74 (0.6-0.92)	0.91 (0.74-1.12)	0.83 (0.68-1.02)	0.91 (0.69-1.2)
		Low PM2.5[c]	High PM2.5[c]	
Non-active commuting		1	1	
≤ two seasons of four		1.18 (0.93-1.48)	0.84 (0.65-1.08)	0.70 (0.50-0.98)
> two seasons of four		0.98 (0.80-1.2)	0.79 (0.64-0.96)	0.82 (0.62-1.09)

[a]Adjusted for sex, calendar year, education, smoking, alcohol intake, occupation, neighbourhood mean income, leisure time physical activity, active commuting

[b]Low PM_{10}: ≤9.6 μg/m³; High PM_{10}: >9.6 μg/m³

[c]Low $PM_{2.5}$ ≤5.7 μg/m³; High $PM_{2.5}$: >5.7 μg/m³

Table 2. Hazard ratios (95% CI) for IHD associated with high air pollution levels (vs low) at home address among persons with different exercise/commuting habits.

	Overall model with No interaction effects	Adjusted[a] HRs in different exercise categories		
		Exercise in training clothes		
		Never	≤ once/week	≥ twice/week
Low PM_{10}[b]	1	1	1	1
High PM_{10}[b]	1.22 (0.96-1.56)	1.25 (1.0-1.56)	1.27 (1.03-1.58)	0.61 (0.40-0.93)
Low $PM_{2.5}$[c]	1	1	1	1
High $PM_{2.5}$[c]	0.95 (0.74-1.21)	1.15 (0.92-1.43)	1.16 (0.94-1.44)	0.64 (0.42-0.97)
		Active commuting		
		Non-active commuting	≤ two seasons of four	> two seasons of four
Low PM_{10}[b]		1	1	1
High PM_{10}[b]		1.28 (1.04-1.57)	0.94 (0.68-1.29)	1.16 (0.9-1.5)
Low $PM_{2.5}$[c]		1	1	1
High $PM_{2.5}$[c]		1.23 (1.0-1.51)	0.88 (0.64-1.21)	0.99 (0.76-1.27)

[a]Adjusted for sex, calendar year, education, smoking, alcohol intake, occupation, neighbourhood mean income, leisure time physical activity, active commuting

[b]Low PM_{10}: ≤9.6 μg/m³; High PM_{10}: >9.6 μg/m³

[c]Low $PM_{2.5}$ ≤5.7 μg/m³; High $PM_{2.5}$: >5.7 μg/m³

For peer review only

C: Sensitivity analyses with PM10 and PM2.5 concentrations categorized by tertile limits

Table 3. Hazard ratios (95% CI) for IHD associated with different exercise and commuting habits among persons with different air pollution exposure at home addresses.

Exercise in training clothes	Overall model with no interaction effects	Proportional hazard p[d]-value*	Adjusted[a] HRs in categories of high and low particle exposure					
			Low PM_{10}[b]	Proportional hazard p-value[d]	Moderate PM_{10}[b]	Proportional hazard p-value[d]	High PM_{10}[b]	Proportional hazard p-value[d]
Never	1		1		1			
≤ once/week	1.03 (0.90-1.16)	0.36	0.93 (0.74-1.16)	0.42	1.15 (0.94-1.41)	0.95	0.99 (0.80-1.23)	0.37
≥ twice/week	0.76 (0.62-0.93)	0.09	0.95 (0.68-1.32)	0.54	0.83 (0.60-1.15)	0.16	0.51 (0.34-0.77)	0.86
			Low $PM_{2.5}$[c]		Moderate $PM_{2.5}$[c]		High $PM_{2.5}$[c]	
Never			1		1			
≤ once/week			0.98 (0.78-1.22)	0.67	1.08 (0.88-1.32)	0.60	1.02 (0.82-1.26)	0.48
≥ twice/week			0.91 (0.65-1.27)	0.71	0.83 (0.60-1.14)	0.13	0.54 (0.36-0.82)	0.60

[a]Adjusted for sex, calendar year, education, smoking, alcohol intake, occupation, neighbourhood mean income, leisure time physical activity, and active commuting

[b]Low PM_{10}: ≤9.6 μg/m^3; High PM_{10}: >9.6 μg/m^3

[c]Low $PM_{2.5}$ ≤5.7 μg/m^3; High $PM_{2.5}$: >5.7 μg/m^3

[d]p-value of the Schoenfeld residual test of proportional hazards

For peer review only

Table 4. Hazard ratios (95% CI) for IHD associated with high and moderate air pollution levels (vs low) at home address among persons with different exercise/commuting habits.

	Overall model with no interaction effects	Proportional hazard p-value[d]	Adjusted[a] HRs in categories of high and low particle exposure					
			Exercise in training clothes					
			Never	Proportional hazard p-value[d]	≤ once/week	Proportional hazard p-value[d]	≥ twice/week	Proportional hazard p-value[d]
Low PM_{10}[b]	1		1		1		1	
Moderate PM_{10}[b]	1.04 (0.81-1.34)	0.55	1.05 (0.83 - 1.32)	0.35	1.30 (1.03 1.63)	0.95	0.92 (0.61-1.39)	0.26
High PM_{10}[b]	1.00 (0.69-1.45)	0.99	1.16 (0.87 1.53)	0.13	1.23 (0.93-1.64)	0.13	0.62 (0.37-1.04)	0.56
Low $PM_{2.5}$[c]	1		1		1		1	
Moderate PM_{10}[b]	1.12 (0.88-1.43)	0.94	1.11 (0.89 1.39)	0.73	1.23 (0.98-1.53)	0.73	1.02 (0.67-1.54)	0.30
High $PM_{2.5}$[c]	1.15 (0.79-1.68)	0.13	1.18 (0.89 1.57)	0.05	1.23 (0.92-1.64)	0.04	0.71 (0.42-1.18)	0.22

[a]Adjusted for sex, calendar year, education, smoking, alcohol intake, occupation, neighbourhood mean income, leisure time physical activity, and active commuting
[b]Low PM_{10}: ≤9.6 µg/m^3; High PM_{10}: >9.6 µg/m^3
[c]Low $PM_{2.5}$ ≤5.7 µg/m^3; High $PM_{2.5}$: >5.7 µg/m^3
[d]p-value of the Schoenfeld residual test of proportional hazards

Table 5. Interaction hazard ratios between exercise and air pollution for incident IHD

Exercise in training clothes	Adjusted[a] interaction hazard ratio			
	Benefits of exercise comparing moderate with low particle exposure		Benefits of exercise comparing high with low particle exposure	
	Moderate PM_{10}[b]	Proportional hazard p-value[d]	High PM_{10}[b]	Proportional hazard p-value[d]
Never	1		1	
≤ once/week	1.24 (0.91-1.68)	0.51	1.07 (0.78-1.45)	0.99
≥ twice/week	0.88 (0.55-1.39)	0.58	0.54 (0.32-0.92)	0.81
	Benefits of exercise comparing high with low $PM_{2.5}$[b] exposure		Benefits of exercise comparing moderate with low $PM_{2.5}$[b] exposure	
Never	1		1	
≤ once/week	1.11 (0.82-1.50)	0.97	1.04 (0.77-1.42)	0.87
≥ twice/week	0.92 (0.58-1.46)	0.45	0.60 (0.35-1.02)	0.87

[a]Adjusted for sex, calendar year, education, smoking, alcohol intake, occupation, neighbourhood mean income, leisure time physical activity, and active commuting
[b]Low PM_{10}: ≤9.6 µg/m^3; High PM_{10}: >9.6 µg/m^3
[c]Low $PM_{2.5}$ ≤5.7 µg/m^3; High $PM_{2.5}$: >5.7 µg/m^3
[d]p-value of the Schoenfeld residual test of proportional hazards

STROBE 2007 (v4) Statement—Checklist of items that should be included in reports of *cohort studies*

Section/Topic	Item #	Recommendation	Reported on page #
**Title and abstract**	1	(*a*) Indicate the study's design with a commonly used term in the title or the abstract	1
		(*b*) Provide in the abstract an informative and balanced summary of what was done and what was found	2
**Introduction**			
Background/rationale	2	Explain the scientific background and rationale for the investigation being reported	4
Objectives	3	State specific objectives, including any prespecified hypotheses	4
**Methods**			
Study design	4	Present key elements of study design early in the paper	5
Setting	5	Describe the setting, locations, and relevant dates, including periods of recruitment, exposure, follow-up, and data collection	5
Participants	6	(*a*) Give the eligibility criteria, and the sources and methods of selection of participants. Describe methods of follow-up	5
		(*b*) For matched studies, give matching criteria and number of exposed and unexposed	
Variables	7	Clearly define all outcomes, exposures, predictors, potential confounders, and effect modifiers. Give diagnostic criteria, if applicable	5-6
Data sources/ measurement	8*	For each variable of interest, give sources of data and details of methods of assessment (measurement). Describe comparability of assessment methods if there is more than one group	5-6
Bias	9	Describe any efforts to address potential sources of bias	6
Study size	10	Explain how the study size was arrived at	5
Quantitative variables	11	Explain how quantitative variables were handled in the analyses. If applicable, describe which groupings were chosen and why	5-6
Statistical methods	12	(*a*) Describe all statistical methods, including those used to control for confounding	7
		(*b*) Describe any methods used to examine subgroups and interactions	7
		(*c*) Explain how missing data were addressed	5
		(*d*) If applicable, explain how loss to follow-up was addressed	7
		(*e*) Describe any sensitivity analyses	
**Results**			

For peer review only

Participants	13*	(a) Report numbers of individuals at each stage of study—eg numbers potentially eligible, examined for eligibility, confirmed eligible, included in the study, completing follow-up, and analysed	5
		(b) Give reasons for non-participation at each stage	5
		(c) Consider use of a flow diagram	
Descriptive data	14*	(a) Give characteristics of study participants (eg demographic, clinical, social) and information on exposures and potential confounders	7
		(b) Indicate number of participants with missing data for each variable of interest	8
		(c) Summarise follow-up time (eg, average and total amount)	7
Outcome data	15*	Report numbers of outcome events or summary measures over time	7
Main results	16	(*a*) Give unadjusted estimates and, if applicable, confounder-adjusted estimates and their precision (eg, 95% confidence interval). Make clear which confounders were adjusted for and why they were included	8-11
		(*b*) Report category boundaries when continuous variables were categorized	9, 11
		(*c*) If relevant, consider translating estimates of relative risk into absolute risk for a meaningful time period	
Other analyses	17	Report other analyses done—eg analyses of subgroups and interactions, and sensitivity analyses	9-11
**Discussion**			
Key results	18	Summarise key results with reference to study objectives	12
**Limitations**			
Interpretation	20	Give a cautious overall interpretation of results considering objectives, limitations, multiplicity of analyses, results from similar studies, and other relevant evidence	12-13
Generalisability	21	Discuss the generalisability (external validity) of the study results	13
**Other information**			
Funding	22	Give the source of funding and the role of the funders for the present study and, if applicable, for the original study on which the present article is based	14

*Give information separately for cases and controls in case-control studies and, if applicable, for exposed and unexposed groups in cohort and cross-sectional studies.

Note: An Explanation and Elaboration article discusses each checklist item and gives methodological background and published examples of transparent reporting. The STROBE checklist is best used in conjunction with this article (freely available on the Web sites of PLoS Medicine at http://www.plosmedicine.org/, Annals of Internal Medicine at http://www.annals.org/, and Epidemiology at http://www.epidem.com/). Information on the STROBE Initiative is available at www.strobe-statement.org.

For peer review only
